# Breaching the Fortress: Photochemistry of DNA-Caged Ag_10_^6+^

**DOI:** 10.1021/acs.jpcb.3c06358

**Published:** 2023-12-06

**Authors:** Caleb J. Setzler, Caleb A. Arrington, David Lewis, Jeffrey T. Petty

**Affiliations:** † Department of Chemistry, Wofford College, Spartanburg, South Carolina 29303, United States; ‡ Department of Chemistry, Furman University, Greenville, South Carolina 29163, United States

## Abstract

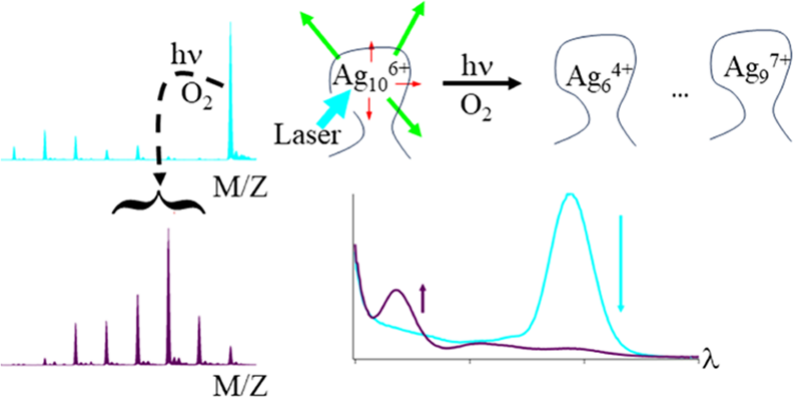

A DNA strand can encapsulate a silver molecule to create a nanoscale, aqueous stable chromophore. A protected cluster that strongly fluoresces can also be weakly photolabile, and we describe the laser-driven photochemistry of the green fluorophore C_4_AC_4_TC_3_GT_4_/Ag_10_^6+^. The embedded cluster is selectively photoexcited at 490 nm and then bleached, and we describe how the efficiency, products, and route of this photochemical reaction are controlled by the DNA cage. With irradiation at 496.5 nm, the cluster absorption progressively drops to give a photodestruction quantum yield of 1.5 (±0.2) × 10^–4^, ∼10^3^× less efficient than fluorescence. A new λ_abs_ = 335 nm chromophore develops because the precursor with 4 Ag^0^ is converted into a group of clusters with 2 Ag^0^ – Ag_6_^4+^, Ag_7_^5+^, Ag_8_^6+^, and Ag_9_^7+^. The 4–7 Ag^+^ in this series are chemically distinct from the 2 Ag^0^ because they are selectively etched by iodide. This halide precipitates silver to favor only the smallest Ag_6_^4+^ cluster, but the larger clusters re-develop when the precipitated Ag^+^ ions are replenished. DNA-bound Ag_10_^6+^ decomposes because it is electronically excited and then reacts with oxygen. This two-step process may be state-specific because O_2_ quenches the red luminescence from Ag_10_^6+^. However, the rate constant of 2.3 (±0.2) × 10^6^ M^–1^ s^–1^ is relatively small, which suggests that the surrounding DNA matrix hinders O_2_ diffusion. On the basis of analogous photoproducts with methylene blue, we propose that a reactive oxygen species is produced and then oxidizes Ag_10_^6+^ to leave behind a loose Ag^+^-DNA skeleton. These findings underscore the ability of DNA scaffolds to not only tune the spectra but also guide the reactions of their molecular silver adducts.

## Introduction

Clusters of silver are distinct from their bulk and nanoparticle forms because they have discrete and widely spaced electronic states and can efficiently fluoresce.^1,2^ As alternatives to organic dyes, these noble metal chromophores are promising optical labels and sensors.^3−8^ However, bare silver clusters are inherently unstable and reactive, but they gain chemical resilience when passivated with ligands.^9−11^ For example, polymers such as polyacrylate can trap multinuclear silver clusters, preventing agglomeration to larger nanoparticles and precipitation as insoluble silver salts.^12^ Such polymeric ligands preserve the distinctive electronic structure of silver clusters, resulting in aqueous stable chromophores. Biopolymers also encapsulate silver molecules, as illustrated by single-stranded oligonucleotides that protect their cluster adducts in harsh aqueous and biological environments.^13−16^ DNA–silver conjugates are recognized by their strong emission and diverse spectra, and one notable application of these chromophores is in biological imaging.^17−23^ Here, conjugates with far-red and near-infrared luminescence can be identified more confidently because the endogenous background is lower relative to the visible region.^24−27^ The cluster spectra span the violet to near-infrared range and can be controlled by the DNA host.

DNA is a scaffold that both protects and forms specific cluster adducts. A single-stranded oligonucleotide folds around a cluster, creating a binding site framed by the nucleobases. These coordination environments can encode the cluster spectra by controlling three factors. First, short stands limit the cluster size.^28^ An oligonucleotide with 10–30 nucleobases strongly binds and locally concentrates silvers to form clusters with 6–30 silvers.^29,30^ Second, a DNA sequence prescribes the set of nucleobase ligands that collectively chelate a cluster.^31−35^ The natural nucleobases and their derivatives bind silvers with varying affinities; therefore, a pattern of nucleobases can be chosen to form a specific cluster. Third, DNA tracts assemble and provide a three-dimensional framework for the cluster.^7,36−40^ These segments of a longer DNA strand can be manipulated to control the cluster spectra, brightness, and quenching. These binding sites not only program the spectra but also guide the chemistry of their silver cluster adducts. For instance, a strand designed from a DNA/silver cluster crystal structure hosts two clusters: a red-emitting Ag_14_^8+^ or a green-emitting Ag_11_^7+^.^41^ A specific adduct is favored by using standard redox reagents: Ag_14_^8+^ can be oxidized to Ag_11_^7+^ using MnO_4_^–^, and Ag_11_^7+^ can be reduced back to Ag_14_^8+^ using H_2_ and added Ag^+^. In this study, we investigated the photofragmentation of a DNA-bound silver cluster.

The oligonucleotide C_4_AC_4_TC_3_GT_4_ is a typical DNA scaffold because it preferentially forms the fluorescent Ag_10_^6+^.^7,42^ The DNA sequence and structure set the foundation for the cluster coordination site. The cytosines are organized into tracts and chelate silvers via their nitrogen and oxygen heteroatoms.^36,37,43^ These tracts are interspersed with nucleobases with distinct roles. Along with the cytosine tracts, the adenine and guanine control the cluster spectra, while the thymine is more inert because it can be enzymatically excised without altering the cluster spectra. Fluorescence anisotropy studies suggest that it acts like a hinge, allowing the strand to fold around Ag_10_^6+^. While Ag_10_^6+^ efficiently fluoresces upon excitation at 490 nm (2.5 eV), this electronic energy can destabilize the cluster.^44,45^ Naked silver clusters can be fragile because their cohesive energy decreases with cluster size, falling to a bond dissociation energy of 1.7 eV for Ag_2_.^46,47^ We examined how the reactivity of energized Ag_10_^6+^ is influenced by its C_4_AC_4_TC_3_GT_4_ host. The cluster is weakly photolabile but bleaches under laser irradiation, and its efficiency was measured using the photodestruction quantum yield. The single Ag_10_^6+^ adduct degrades to multiple photoproducts, which were categorized based on their Ag^0^ and Ag^+^ contents using mass spectrometry and I^–^ etching. Electronically excited Ag_10_^6+^ degrades because it reacts with oxygen, and the reaction pathway was followed using quenching of the red cluster luminescence. C_4_AC_4_TC_3_GT_4_/Ag_10_^6+^ is a supramolecular chromophore, and we discuss how the DNA host tempers the photoreaction and controls the photoproducts of its cluster adduct.

## Methods

C_4_AC_4_TC_3_GT_4_ (Integrated DNA Technologies) was purified by the manufacturer with standard desalting and hydrated to ∼1 mM concentration in water. The concentrations were measured based on the nearest-neighbor approximation.^48^ AgNO_3_ (AlfaAesar, Premion) and NaBH_4_ (Thermo Scientific, 98.5%) were used as received and dissolved in water with concentrations of ∼30 mM. DNA/silver cluster complexes were synthesized by diluting the DNA to 30 μM in a 10 mM pH 7 cacodylate buffer followed by the addition of AgNO_3_ in an 8 Ag^+^:DNA ratio. The resulting solution was heated to 80 °C for 5 min and then cooled. NaBH_4_ was added in a 4 BH_4_^–^:DNA ratio. Typically, such reaction conditions yield a range of clusters, so 8 Ag^+^ was chosen to inhibit larger species.^49^ Also, specific clusters can be favored with sufficient time and by oxygen, which can selectively oxidize clusters to favor specific chromophores.^12,41,50^ In these studies, the DNA/Ag_10_^6+^ conjugate was synthesized using 400 psi O_2_ for a minimum of 2 h, and the resulting cluster spectrum does not change with longer exposure.^49^ The resulting complexes are stable in solutions diluted up to ∼10,000-fold volumes of buffer and do not disproportionate.^51^ These samples were then irradiated with a continuous-wave argon-ion laser tuned to 496.5 nm. The laser irradiance was regulated using neutral density22 filters. The reaction was monitored using UV–visible (Cary 50, Varian) and fluorescence (Fluoromax-3, Jobin-Yvon HORIBA) spectroscopy.

The lifetimes of the metastable Ag_10_^6+^ clusters were measured by passing the argon laser through an acousto-optical modulator with a fixed-frequency driver (MT200-A0.5-VIS, AA Optoelectronic) to modulate laser intensity. The fixed-frequency driver provided a 1 kHz square waveform with 50% duty cycle and peak-to-peak amplitude of 0–1 V by a function generator (33250A, Agilent). Laser pulses were focused to a 3.5 μm spot inside a 3 mm × 3 mm quartz cell (3.3–45-Q-3, Starna Cells). Luminescence was measured at a right angle by an avalanche photodiode (SPD-050, Micro Photonic Devices) through a 730 nm LP filter. Pulses from the detector were processed by a counter/timer card (PCI-6612, National Instruments) operating in multichannel scaling mode. Laser on/off cycles populated excited states that radiatively decay on microsecond time scales. This luminescence is modeled by a single exponential function to determine the lifetime.

Stern–Volmer quenching analysis was performed by purging solutions in nitrogen, oxygen, or air for 5 min before capping and measuring the lifetime of the long-lived state. Quenching coefficients for excited states are described by^52^
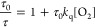
1where τ_0_ is the lifetime of the excited state following a nitrogen purge, τ is the lifetime of the excited state at a given O_2_ concentration, [O_2_] is the molar concentration of oxygen in solution, and *k*_q_ is the quenching constant. Oxygen concentrations were calculated by the Henry’s law constant of 1.2 × 10^–5^ mol Pa^–1^ m^–3^.^53^

Mass spectra were recorded with a Q-TOF G2-S mass spectrometer (Waters). DNA–silver clusters were purified and desalted by dilution 100 times in a 10 mM ammonium acetate solution before dialysis with 2 kDa cutoff filters. The resulting solutions were diluted to 2 μM oligonucleotide in 10 mM ammonium acetate for analysis by mass spectrometry. Analyte solutions were infused with a syringe pump with a flow rate of 30 μL/min. Mass spectra were collected in negative ion mode with a capillary voltage of −2.7 kV, a sampling cone voltage of −15 V, an extraction cone voltage of 10 V, a cone gas flow of 45 L/h, and a desolvation gas flow of 450 L/h. The source and desolvation temperatures were 80 and 150 °C, respectively. Sodium iodide aggregates in the 400–2000 *m*/*z* range provided the mass calibration profile.

Isotopologue distributions derived from the mass spectra determined the cluster size and charge. As a reference, model distributions were derived from the formula C_169_H_222_N_53_O_110_P_17_Ag_10_ for the fully protonated oligonucleotide. These were largely controlled by the nearly equal proportions of ^107^Ag (51.8%) and ^109^Ag(48.2%). H^+^ were subtracted to account for the overall DNA charge and the charge of the Ag_10_ adduct. Experimental data were converted to a series of (*x*,*y*) pairs corresponding to the *M*/*Z* and intensity values with the latter normalized using the most abundant peak in the distribution. The differences between the predicted and observed *M*/*Z* values and intensities are reported as average absolute values over the full breadth of a given distribution. Each distribution typically had ∼15 peaks, and each DNA–cluster complex had 3–4 ions that were analyzed. An example data set for (C_4_AC_4_TC_3_GT_4_/Ag_10_^6+^)^−4^, (C_4_AC_4_TC_3_GT_4_/Ag_10_^6+^)^−5^, (C_4_AC_4_TC_3_GT_4_/Ag_10_^6+^)^−6^, and (C_4_AC_4_TC_3_GT_4_/Ag_10_^6+^)^−7^ is provided in Tables S2 and S3. A home-written program analyzed the full set of data.

Photodissociation quantum yields (Φ_d_) were measured using an argon-ion laser. The total duration and power of irradiation were set so that absorbance would drop by no more than 0.2 to keep the fraction of incident photons absorbed nearly consistent over the experiment. Absorption spectra for the ∼25 μM solutions of DNA–silver clusters (*A* ≈ 1.2) were collected before and after irradiation. The samples in quartz cuvettes were stirred. Changes in the concentration of silver clusters were calculated with the extinction coefficient ϵ = 48,000 M^–1^ cm^–1^ at 488 nm.^7^ Photodissociation quantum yields are calculated by
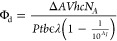
2where Δ*A* is the change in absorbance at the peak absorbance, *V* is the volume of the solution, *h* is Planck’s constant, *c* is the speed of light, *N*_*A*_ is Avogadro’s number, *P* is the power output of the laser, *t* is the total time of irradiance, *b* is the path length of the cell, λ is the wavelength of the incident photons, and *A*_l_ is the average absorbance of the sample at 496.5 nm. An example calculation is provided in Table S1. The procedure for measuring photodissociation quantum yield was verified using ferrioxalate actinometry.^54^

## Results

### Spectra and Photochemistry

**Figure 1:**
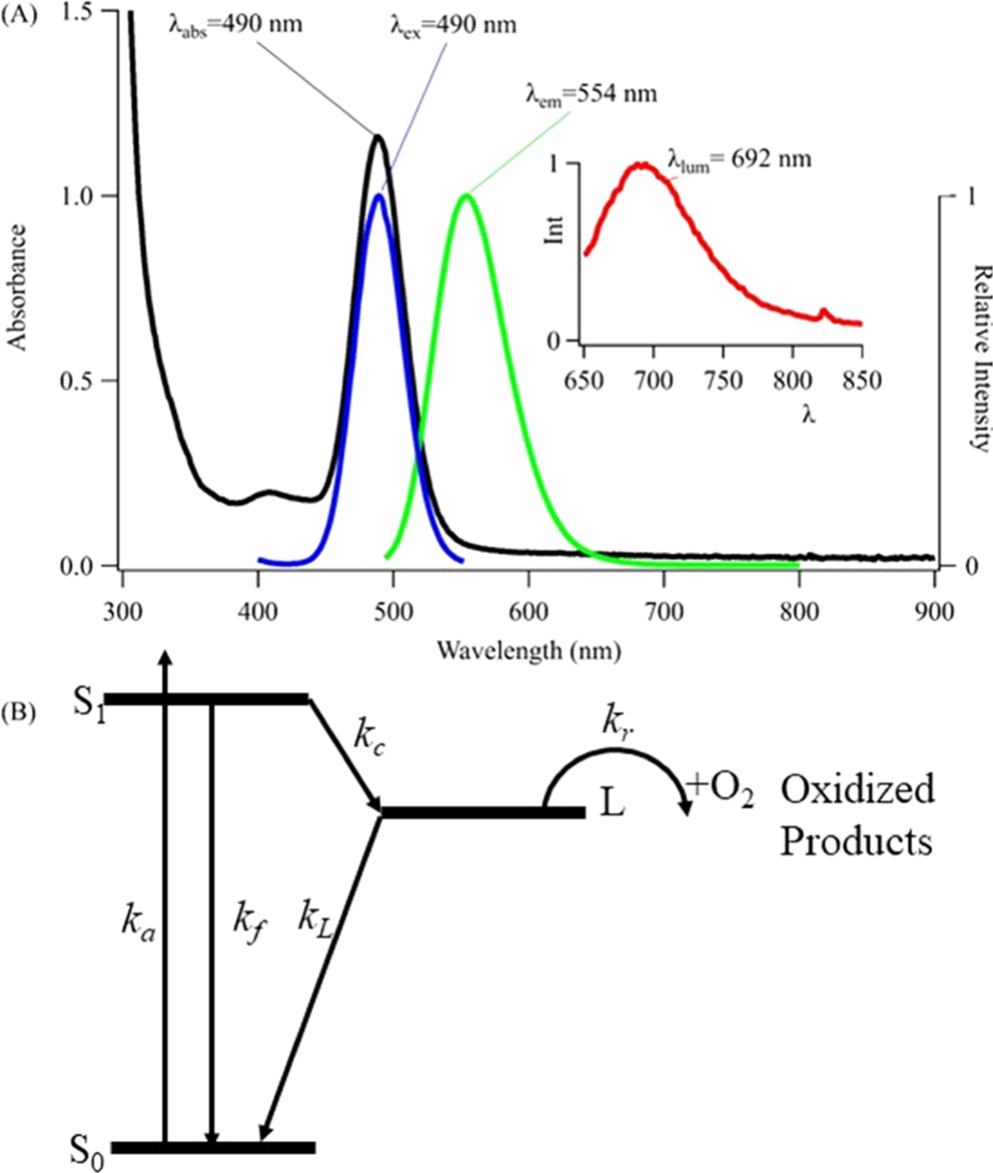
Spectroscopy and photochemistry: (A) Absorption (black-left axis) and excitation(blue-)/emission (green-right axis) spectra of Ag_10_^6+^/C_4_AC_4_TC_3_GT_4_. Coincident absorption and excitation spectra suggest that this is the dominant chromophore. The inset shows the λ ∼ 700 nm luminescence acquired in an ethylene glycol/buffer glass at 77 K. This red luminescence is ∼300× weaker than the green emission. (B) Energy level diagram for the Ag_10_^6+^/C_4_AC_4_TC_3_GT_4_ chromophore. The cluster is first excited from its ground S_0_ state to its green-emissive S_1_ state (*k*_a_) and then either relaxes radiatively and nonradiatively back to S_0_ (*k*_f_) or crosses to its red-luminescent L state (*k*_c_). The metastable cluster in L can relax either radiatively or nonradiatively back to S_0_ (*k*_L_) or react with O_2_ (*k*_r_). The general L label is used because studies of the electronic configuration are ongoing.

The C_4_AC_4_TC_3_GT_4_-bound cluster has two key features that underlie its photochemistry. First, this green-emitting fluorophore efficiently absorbs light due to its high molar absorptivity (ε) of 48,000 M^–1^ cm^–1^ (Figure 1A). A fraction of its electronic energy drives the photochemistry. Second, it luminesces at λ ∼ 700 nm, which we attribute to a metastable L state situated between its ground S_0_ and green-emissive S_1_ states (Figure 1A(inset),B).^40,44,55^ Three observations support this assignment: (1) The red luminescence and green fluorescence have the same λ_ex_ = 490 nm, suggesting that S_0_ → S_1_ excitation precedes and feeds the L state (Figure S1). (2) The red luminescence is relatively weak, even in a cryogenic matrix, implying that the radiative L → S_0_ transition is disfavored. This inefficiency is also observed in the related C_4_AC_4_TC_3_GT/Ag_10_^6+^ chromophore. (3) The red luminescence slowly builds and decays with an ∼80 μs lifetime, ∼10^4^× slower than the fluorescence decay (Figure S2). This sluggish response again suggests that the L ↔ S_0_/S_1_ transitions are disfavored. This metastable state is a potential reaction gateway because it stores electronic energy, and its long lifetime favors collisional, bimolecular reactions.

### Photodegradation Efficiency

**Figure 2:**
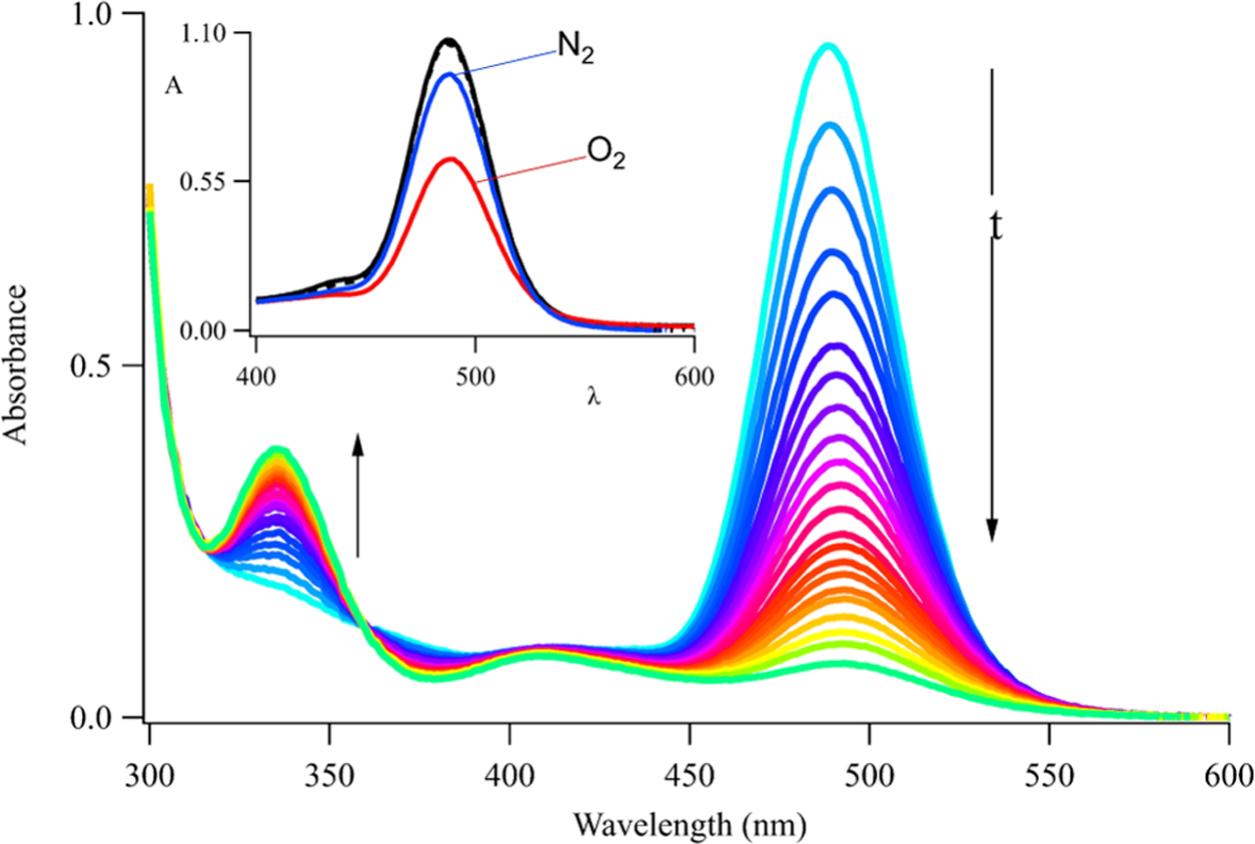
Photodegradation efficiency: A time series showing that the λ_abs_ = 490 nm Ag_10_^6+^ cluster bleaches (down arrow) with irradiation to yield a new cluster with λ_abs_ = 335 nm (up arrow) over 29 min using 3 mW at 490 nm. The inset highlights more efficient photodestruction in solutions purged with oxygen (red) versus nitrogen (blue). The two solutions have coincident starting spectra (black).

The degradation of C_4_AC_4_TC_3_GT_4_-Ag_10_^6+^ was quantified by using the photodestruction quantum yield (Φ_d_), which represents the number of clusters destroyed per photoexcitation (Figure 2). The 490 nm absorption band progressively drops during irradiation, yielding Φ_d_ = 1.5 (±0.2) × 10^–4^. Several observations provide insight into this photoreaction. Consistent Φ_d_ measurements over the duration of an experiment indicate that photoproducts do not impede degradation (Table S1). Photoreaction ceases upon halting irradiation, indicating that the cluster degrades because it is electronically excited (Figure S3). The reaction efficiency remains consistent across a 10-fold range of irradiances, suggesting that a single photon drives the photoreaction. Solvent isotope effects using H_2_O and D_2_O are negligible, which suggests that the DNA host insulates the cluster from the bulk solvent (Figure S3).^56^ The 490 nm cluster bleaches in a first-order manner, indicating that a high photon flux inefficiently degrades the cluster (Figure S4). We next focus on the λ = 335 nm species that replaces the bleached cluster (Figure 2). Because bleaching and growth are correlated, an extinction coefficient of the new chromophore is estimated to be ε = 13,000 M^–1^ cm^–1^ through mass balance considerations. This value aligns with other small silver clusters.^31,57^

### Multivalent Strand

The C_4_AC_4_TC_3_GT_4_ template forms adducts with distinct amounts of Ag^0^ and Ag^+^, which were quantified by using electrospray ionization mass spectrometry. Two features of the DNA host were leveraged for this analysis.^29,58^ First, C_4_AC_4_TC_3_GT_4_ is a synthetic and monodisperse polymer, so the total number of silver adducts can be measured. Second, the phosphodiester backbone acts like a buffer because it adapts to the silver cluster. The Ag^+^ in a cluster partially neutralize the cumulative negative charge of the phosphates, consequently reducing the number of H^+^ acquired during desolvation.^29^ This electrostatic balance of Ag^+^ and H^+^ is illustrated by unligated vs ligated forms of (C_4_AC_4_TC_3_GT_4_)^−4^ (Tables S2 and S7). When comparing (C_4_AC_4_TC_3_GT_4_)^−4^ alone with (C_4_AC_4_TC_3_GT_4_)^−4^ with an Ag_10_ adduct, their respective chemical formulas are C_169_H_218_N_53_O_110_P_17_ and C_169_H_212_N_53_O_110_P_17_Ag_10_. As the underlining indicates, the DNA-Ag_10_ conjugate has 6 fewer H^+^ while still maintaining its net −4 charge. This overall charge balance suggests that these 6 H^+^ are replaced by 6 Ag^+^, so the cluster is an oxidized Ag_10_^6+^ adduct. Each DNA/cluster ion displays an isotopologue distribution primarily due to nearly equal proportions of ^107^Ag and ^109^Ag, and these can be fit with ±1 H^+^ precision (Figure S5 and Tables S2 and S7). Ag^+^ counted in this manner align with solution measurements of the net cluster oxidation state using XANES spectra.^51^ Subtracting these Ag^+^ counts from the total number of silvers gives the number of Ag^0^, which controls the cluster photochemistry.

**Figure 3:**
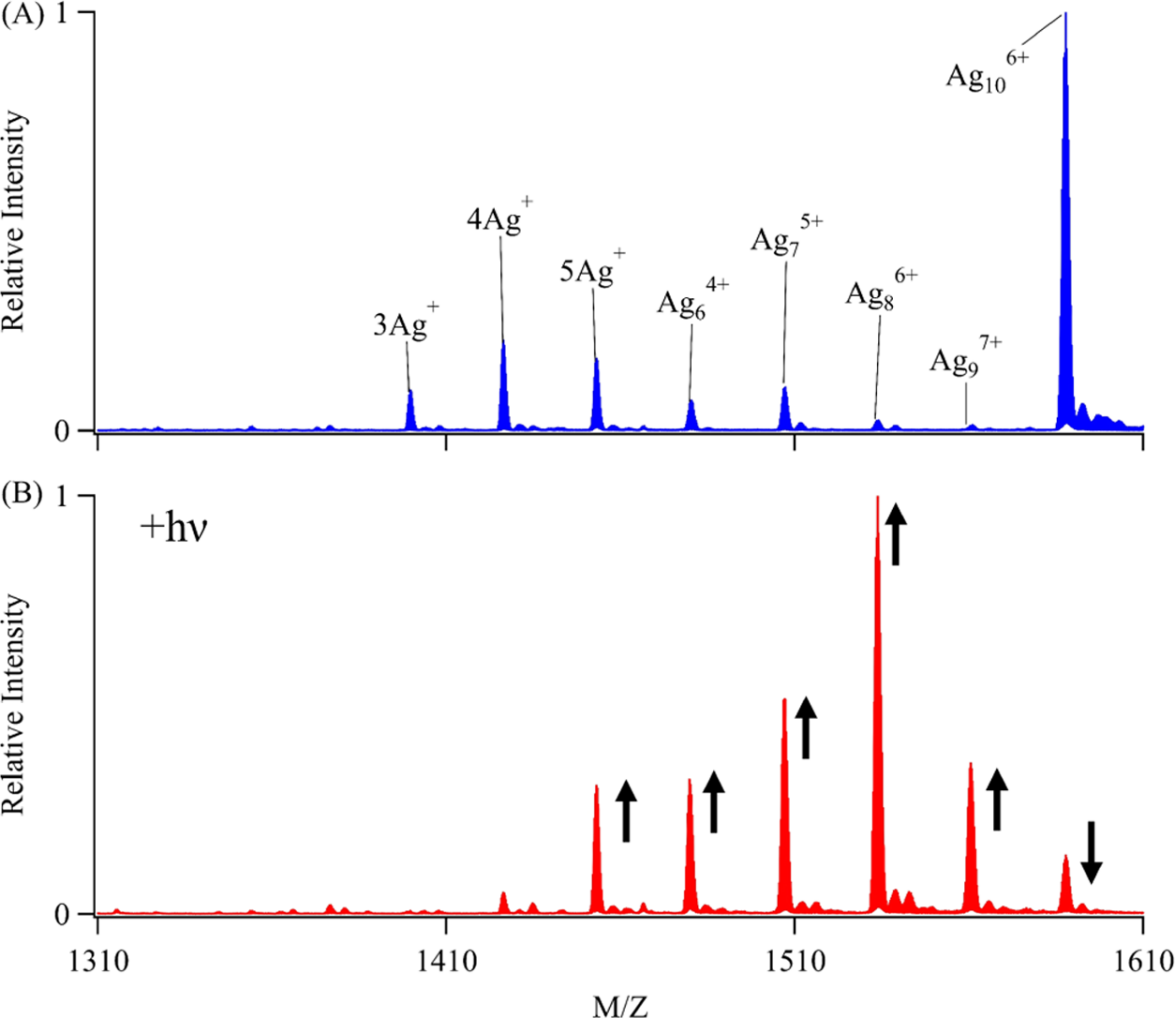
Multivalent strand: Mass spectra of (C_4_AC_4_TC_3_GT_4_/Ag_10_^6+^)^−4^ before (A) and after (B) irradiation at 496.5 nm. Ag_10_^6+^ dominates before irradiation but converts mainly to a set of 2 Ag^0^ clusters (Ag_9_^7+^, Ag_8_^6+^, Ag_7_^5+^, and Ag_6_^4+^) and a smaller amount of purely Ag^+^ adducts (with 5 and 6 Ag^+^). Arrows describe the photobleaching of Ag_10_^6+^ and the growth of its photoproducts.

Within C_4_AC_4_TC_3_GT_4_, three types of adducts form with 4 Ag^0^, 2 Ag^0^, and only Ag^+^. Among these, Ag_10_^6+^ dominates. It has 4 Ag^0^ like other green-emitting DNA-bound clusters, so this C_4_AC_4_TC_3_GT_4_/Ag_10_^6+^ conjugate is assigned to be the λ_ex_/λ_em_ = 490/550 nm chromophore (Figure 1A).^32^ This cluster is consistently identified in four DNA/Ag_10_^6+^ ions with net −4, −5, −6, and −7 charges, where these net charges vary only because of phosphate protonation (Figure 3A and Tables S2 and S7).^59^ Alongside Ag_10_^6+^, a less abundant set of 2 Ag^0^ clusters develop. Ag_6_^4+^, Ag_7_^5+^, Ag_8_^6+^, and Ag_9_^7+^ are grouped because their 2 Ag^0^ will appear as a single chromophore (Tables S3–S6 and S8). Lastly, a set of 3–5 Ag^+^ also binds with C_4_AC_4_TC_3_GT_4_, and such purely oxidized silver complexes also develop when Ag^+^ alone is combined with C_4_AC_4_TC_3_GT_4_ (Figure S6). Specific complexes were identified by varying the Ag^+^ concentrations and diluting with a 100× volume of buffer to drive off weakly bound adducts. Starting with a relatively high concentration of 8 Ag^+^:DNA, most Ag^+^ are lost to dilution, resulting in 3–4 Ag^+^:DNA complexes. From the opposite perspective, a relatively low initial concentration of 2 Ag^+^:DNA yields a bimodal distribution with strands that accumulate an average of 3 Ag^+^:DNA along with strands that are starved of Ag^+^. Finally, an initial intermediate concentration of 4 Ag^+^:DNA remains relatively stable with dilution. These consistent stoichiometries of 3–4 Ag^+^/DNA align with the stoichiometry in the cluster sample. We now discuss how these three types of Ag^0^/Ag^+^ adducts reorganize when Ag_10_^6+^ photobleaches.

### Ag^0^ Photochemical Toehold

The Ag^0^ in Ag_10_^6+^ can be electronically excited to channel energy into Ag_10_^6+^, and then a fraction of the energized clusters decomposes. The resulting photoproducts were characterized via their optical and mass spectra. Prolonged irradiation at λ = 490 nm bleaches the Ag_10_^6+^ absorption and generates a new λ = 335 nm peak. This new species behaves like a silver cluster, with distinctive spectral features. It fluoresces with λ_ex_/λ_em_ = 335/480 nm, and its blue-shifted spectrum relative to Ag_10_^6+^ supports a smaller but still reduced cluster (Figures 2 and S7B). For example, transient Ag_4_^2+^ and Ag_3_^+^ clusters nascently develop using radiolysis and electrolysis and absorb with λ ∼ 300 nm.^60−63^ Matrix-isolated Ag_2_ clusters also absorb in the violet region at λ ∼ 370 nm with ∼500 nm emission.^3,64^ Likewise, Ag_4_^2+^ trapped in zeolite cavities absorb at ∼330 with ∼550 nm emission.^65^ Hence, these clusters with 2 Ag^0^ generally absorb in the violet region with green emission, as observed when Ag_10_^6+^ is photolyzed.

While the optical spectra identify a single photoproduct with λ = 335 nm, three observations from the mass spectra paint a fuller picture of the photodissociation (Figure 3B). First, the C_4_AC_4_TC_3_GT_4_/Ag_10_^6+^ conjugate is eliminated, firmly establishing this conjugate as the λ_abs_ = 490 nm chromophore. Second, the DNA is unchanged because it is optically transparent and not altered by relatively low-energy 490 nm excitation. Third, the single C_4_AC_4_TC_3_GT_4_/Ag_10_^6+^ precursor disperses into multiple photoproducts: C_4_AC_4_TC_3_GT_4_/Ag_6_^4+^, /Ag_7_^5+^, /Ag_8_^6+^, and/Ag_9_^7+^. All have 2 Ag^0^ and are collectively assigned as the λ_abs_ = 335 nm photoproduct because these complexes are stable and do not dissociate when diluted with 100× and 10,000× volumes of buffer, unlike weakly bound Ag^+^-C_4_AC_4_TC_3_GT_4_ complexes (Figure 2 and compare Figures S8 vs S6). In concert with the growth of 2 Ag^0^ clusters, strands with 5–6 Ag^+^ also develop. Thus, the 4 Ag^0^ in Ag_10_^6+^ are targeted to favor 2 Ag^0^ and purely Ag^+^ adducts.

### Iodide Etching

**Figure 4:**
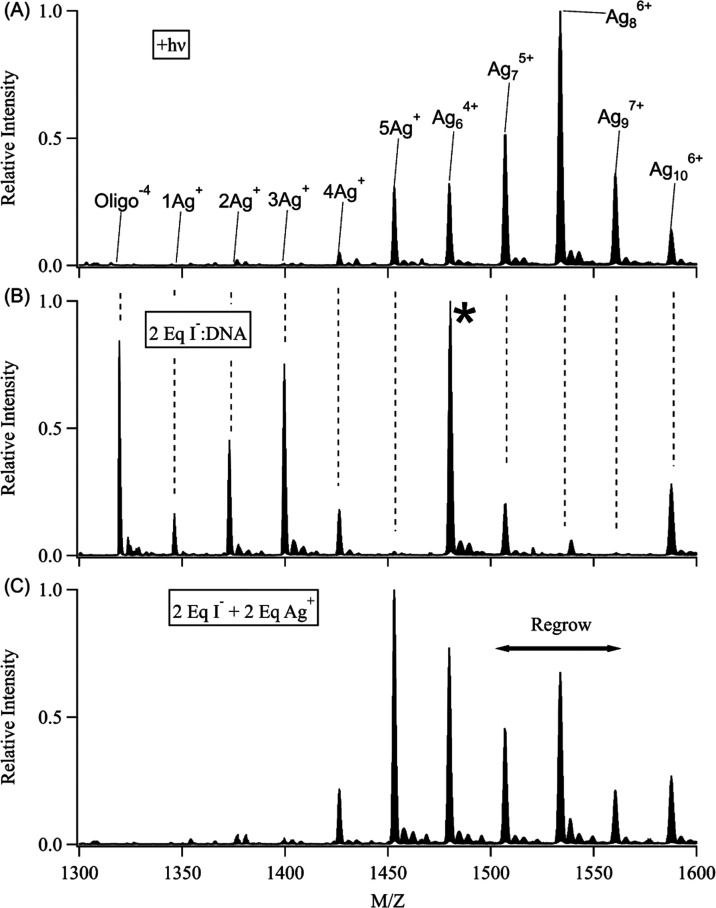
Iodide etching: (A) C_4_AC_4_TC_3_GT_4_/Ag_10_^6+^ after photolysis favors Ag_6_^4+^, Ag_7_^5+^, Ag_8_^6+^, and Ag_9_^7+^. (B) This distribution is pared down to Ag_6_^4+^ with 2 I^–^:DNA as indicated by the *. (C) The larger clusters regrow after adding back 2 Ag^+^:DNA to replace the precipitated silver.

The Ag_6_^4+^, Ag_7_^5+^, Ag_8_^6+^, and Ag_9_^7+^ adducts all have 2 Ag^0^ and appear as the same chromophore, while the 4–7 Ag^+^ in these clusters remain optically silent. Moreover, these Ag^+^ are chemically distinct from Ag^0^ because they selectively react with iodide. This halide binds Ag^+^ ∼ 10^10^ times stronger than DNA, as evident in the optical and mass spectra.^66,67^ When 2 I^–^:DNA are added, the absorption feature at λ_abs_ ≲ 430 nm due to AgI(s) emerges, establishing that silvers are stripped from DNA (Figure S7A).^68^ I^–^ targets and eliminates Ag^+^ from the larger clusters to favor the smallest Ag_6_^4+^ cluster, but the larger clusters reassemble when the precipitated Ag^+^ is replaced (Figure 4). The redistributions shown in the mass spectra are relative changes, while the absorption and fluorescence spectra show overall changes. The cluster absorption and fluorescence drop with I^–^ and do not recover by adding Ag^+^, implying that I^–^ reacts with Ag^0^ to a lesser extent relative to that of Ag^+^ (Figure S7B). These changes are correlated with halide affinity because the reaction is hindered with Br^–^, and no reaction is observed with Cl^–^ (Figure S9). Consequently, iodide etching implies that the photoproducts of Ag_10_^6+^ have modular Ag^0^ and Ag^+^ constituents. Next, we consider how Ag^0^ in Ag_10_^6+^ selectively photodegrade.

### Oxygen-Mediated Destruction

When the Ag^0^ in Ag_10_^6+^ are electronically excited, the ensuing photoreaction is enhanced by oxygen (Figures 2 and S10). For samples saturated with O_2_ vs air, Ag_10_^6+^ decomposes ∼2× more efficiently with a quantum yield (Φ_d_) of 3 (±0.2) × 10^–4^. Conversely, photodestruction is hindered by using N_2_ purging. Luminescence quenching pinpoints that O_2_ may react via a specific cluster electronic state. While it does not perturb the 1.9 ns green emission from S_1_, oxygen quenches the 80 μs red luminescence from the L state with a rate constant of 2.3 (±0.2) × 10^6^ M^–1^ s^–1^ (Figure S11). This rate constant is almost 10^3^× below a diffusion-limited value, considering diffusion coefficients of 2 × 10^–5^ cm^2^/s for O_2_ and 1.4 × 10^–6^ cm^2^/s for the DNA/Ag_10_^6+^, a collision radius of 10 Å, and statistical spin effects, assuming a quenching from a triplet state.^44,69^

These oxygen-dependence and quenching studies suggest that the electronic energy in Ag_10_^6+^ dissipates by electronic relaxation followed by reaction with O_2_. The state energy diagram more specifically describes these two steps, and we now discuss how the efficiencies of the electronic transitions and reactions are linked to the overall photodestruction quantum yield.^69^ Ag_10_^6+^ is initially excited to its S_1_ state and then either relaxes back to S_0_ (*k*_f_) or crosses to its neighboring L state (*k*_c_). This crossing efficiency is
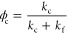
3Next, the population in L relaxes to S_0_ (*k*_L_) or is quenched by/reacts with O_2_ (*k*_r_). The associated reaction efficiency is
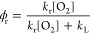
4The state efficiencies ϕ are related to the observed Φ_d_ by the formula^69,70^
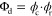
5Using the previously measured ϕ_c_ = 0.05 and *k*_f_ = 5.3 × 10^8^/s, the associated *k*_c_ is 2.5 × 10^6^/s, which is similar to theoretical predictions of intersystem crossing rates.^7,71^ In addition, the net quantum yield Φ_d_ = 1.5 × 10^–4^ gives ϕ_r_ = 0.003.^7,69,72,73^ Using  = 12.5 kHz and [O_2_]_ambient_ = 0.26 mM in water, the reaction rate constant is 1.5 × 10^5^ M^–1^ s^–1^, ∼10× slower than the Stern–Volmer quenching constant. This inefficiency suggests that the photochemical reaction of O_2_ with the DNA-silver complex contributes to quenching. The Ag_10_^6+^ would bleach in a pseudo-first-order manner because the reaction is inefficient and the O_2_ concentration is relatively high (Figure S4).

To further investigate how Ag_10_^6+^ photodecomposes, the roles of light excitation and then reaction with O_2_ were decoupled using methylene blue.^74^ This dye absorbs with λ_abs_ = 630 nm, ensuring that the cluster remains in its ground S_0_ state. Following excitation, the methylene blue intersystem crosses to its triplet state with a 50 μs lifetime. This electronically excited dye is an oxidizing agent that emulates direct photoexcitation of the cluster: the 490 and 335 nm absorptions of Ag_10_^6+^, respectively, decrease and increase, and the same distribution of 2 Ag^0^ and Ag^+^ complexes develops (Figure S12).^56,75^ Therefore, Ag_10_^6+^ can be decomposed via an exogenous oxidizing agent, and we suggest that electronically excited methylene blue and reactive oxygen species produce similar outcomes.

## Discussion

C_4_AC_4_TC_3_GT_4_ is a polymer that selectively forms and specifically binds Ag_10_^6+^, as signified by single λ_abs_/λ_em_ = 490/550 nm electronic transitions in the optical spectra and by a dominant C_4_AC_4_TC_3_GT_4_/Ag_10_^6+^ conjugate in the mass spectra. This coordination site also functions as a cage, and we discuss how it regulates and guides the photochemistry of its trapped Ag_10_^6+^ adduct.

### Protective Shell

The state energy diagram is a map that describes how electronically excited Ag_10_^6+^ dissipates its energy and reacts with oxygen (Figure 1B). We explore how the C_4_AC_4_TC_3_GT_4_ host guides these changes. The photoreaction has two bottlenecks that are both associated with the L state. First, this state is populated sequentially: S_0_ → S_1_ excitation was followed by S_1_ → L crossing. The low ϕ_c_ ∼ 0.05 for S_1_ → L aligns with other strongly fluorescent DNA-bound silver clusters that favor radiative S_1_ → S_0_ relaxation.^26,40,73^ Weak S_1_ → L transitions may be intersystem crossing because frontier orbitals of the silvers and their ligated nucleobases can mix.^71^ Second, the L state has an ∼80 μs lifetime, so an energized, long-lived form of Ag_10_^6+^ could react with O_2_. In support, luminescence from this metastable Ag_10_^6+^ is quenched by O_2_, but the *k*_SV_ = 2.3 × 10^6^ M^–1^ s^–1^ is ∼10^3^× slower than diffusion-controlled. Correspondingly, the overall photoreaction is inefficient with ϕ_r_ ∼ 0.003, and the related photodestruction rate constant of 1.5 × 10^5^ M^–1^ s^–1^, 10× slower than the quenching rate constant. To better understand these inefficiencies, we consider how oxygen degrades Ag_10_^6+^.

The photoreaction converts the 4 Ag^0^ in Ag_10_^6+^ to mainly the 2 Ag^0^ products Ag_6_^4+^, Ag_7_^5+^, Ag_8_^7+^, and Ag_9_^7+^. This 4 Ag^0^ → 2 Ag^0^ conversion could follow two plausible routes involving oxygen.^76^ One, metastable Ag_10_^6+^ can transfer its energy to O_2_ by leveraging the 1.8 eV gap between the L and S_0_ states to excite ^3^O_2_ → ^1^O_2_, which has a smaller energy gap of 1 eV.^69^ This electronically excited O_2_ could then oxidize the cluster, a favored reaction for silver molecules.^12,77^ Two, the excited cluster can transfer an electron to reduce O_2_, and the reactive O_2_^–^ yields a cascade of reactive oxygen species.^78,79^ In either scenario, O_2_ must navigate through DNA to oxidize the cluster.

C_4_AC_4_TC_3_GT_4_ is a polymer that folds to encapsulate its Ag_10_^6+^ adduct. A protective barrier is suggested by the solvent dependence of the Ag_10_^6+^ + O_2_ photoreaction. D_2_O was used because it lengthens the lifetime and thus enhances the reactivity of ^1^O_2_ relative to H_2_O.^56^ However, Ag_10_^6+^ degrades with similar efficiencies in these two solvents, suggesting that the C_4_AC_4_TC_3_GT_4_ host incarcerates and shields Ag_10_^6+^ from oxygen in the bulk solvent. Exogenous agents other than O_2_ also degrade Ag_10_^6+^. Methylene blue was used because it can be electronically excited to yield a strong oxidizing agent. It yields the same products as when the cluster is photoexcited, suggesting that methylene blue and oxygen are both exogenous agents that must penetrate the DNA to degrade the cluster. Prior studies have revealed that proteins also hinder oxygen diffusion.^80^ Tryptophan quenching is position-dependent, with surface/solvent-exposed residues relaxing ∼1000 times faster than buried ones. This observation aligns with duplex DNA that transiently opens and allows O_2_ to quench intercalated methylene blue.^81^ Additionally, a DNA-bound silver cluster is quenched more efficiently by O_2_ when the phosphodiester backbone is split.^40^ This protection is also observed for rotoxanes and cyclodrexins that encapsulate and protect organic dyes from photooxidation.^82,83^ A DNA is analogous to these synthetic scaffolds because its sequence and structure can be tuned and controlled.

### Loose Cage

C_4_AC_4_TC_3_GT_4_ further regulates the photoreaction by controlling the types of photoproducts. The single Ag_10_^6+^ precursor is downsized to Ag_6_^4+^, Ag_7_^5+^, Ag_8_^6+^, and Ag_9_^7+^, which all have 2 Ag^0^ and appear as the single λ_abs_ = 335 nm chromophore. Theoretical studies have identified a Ag_4_^2+^ cluster with green emission, and such a core in the DNA/Ag_10_^6+^ complex might photodegrade in a stepwise manner to release 2 Ag^0^, 2 Ag^0^ + Ag^+^, and 2 Ag^0^ + 2 Ag^+^.^71^ This scenario could yield the observed distribution of products. The 2 Ag^0^ that remain in the photoproducts are consistent with the even numbers of Ag^0^ in a wide range of DNA-bound silver clusters, whose electronic stability is dictated by their shape.^30,32,51,84^ Silver clusters with 2 Ag^0^ have been previously identified in dC_12_/Ag_6_^4+^ and peptide/Ag_3_^+^ complexes.^29,85^ Thus, Ag_10_^6+^/C_4_AC_4_TC_3_GT_4_ mimics other noble metal clusters because the electronic stability of its photoproducts guides photodissociation.^86−88^

DNA-bound silver clusters are oxidized, but these adducts can be atomically imprecise because the number of Ag^+^ varies. For example, DNA-bound Ag_10_^6+^, Ag_12_^8+^, and Ag_11_^7+^ are all green emitters, presumably because they have a common Ag_4_^0^ core.^7,28,41,89^ Here, the Ag_6_^4+^, Ag_7_^5+^, Ag_8_^6+^, and Ag_9_^7+^ photoproducts all have 2 Ag^0^ but 4–7 Ag^+^. We suggest that these Ag^+^ are remnants when the Ag^0^ in Ag_10_^6+^ are oxidized. This oxidation thus upsets the balance of Ag^+^ and Ag^0^ in the preexisting Ag_10_^6+^ binding site, leading to a less stable and “looser” complex. In support, Ag^+^ are removed when I^–^ degrades the larger Ag^+^-rich clusters, leaving behind the smallest Ag_6_^4+^ cluster. However, the stripped and precipitated Ag^+^ can be replaced, and the larger clusters reform. This ebb and flow suggests that Ag^+^ is weakly bound and modular because they are distinct from Ag^0^. A disconnect between the two types of silver is supported by the repeated sequences (C_2_A)_6_ and (C_2_A)_8_ that respectively form Ag_10_^6+^ and Ag_12_^8+^ - identical green-emitting fluorophores due to their 4 Ag^0^.^28^ However, (C_2_A)_8_ has two additional Ag^+^that fill coordination sites in this longer scaffold. Also, silver clusters with identical spectra have the same numbers of Ag^0^ but different amounts of Ag^+^ due to their different DNA sequences and cluster binding sites.^32,55^ For example, three DNA-bound clusters with 13, 14, and 16 total silvers have the same number of Ag^0^ and appear as a single near-infrared emitting chromophore.^55^ From a structural perspective, Ag^0^ and Ag^+^ constituents segregate in Ag_10_^6+^/DNA complexes, as EXAFS spectra reveal a metal-like core surrounded by a Ag^+^-DNA shell.^51,90^

We propose that a lock with a loose key model can describe the photochemistry of Ag_10_^6+^/C_4_AC_4_TC_3_GT_4_. Ag_10_^6+^ precisely fits within C_4_AC_4_TC_3_GT_4_, but irradiation and oxygen etch away Ag^0^ to leave the Ag^+^-DNA skeleton around the 2 Ag^0^ chromophore. These more oxidized clusters loosely fit the preexisting coordination site, as suggested by their etching with I^–^. Understanding these “poor” binding sites will help advance studies of DNA-guided silver cluster photochemistry.

We return to our original thesis that silver clusters behave like molecules. Comparatively, the photochemistry of Ag_10_^6+^/C_4_AC_4_TC_3_GT_4_ bears similarities to fluorescein, a typical organic dye with widely spaced valence electronic states.^77,91^ Both efficiently fluoresce and are weakly photolabile.^92^ Both react from a metastable electronic state produced indirectly via the strongly emissive state, eventually degrading via oxygen.^77,93^ However, Ag_10_^6+^/C_4_AC_4_TC_3_GT_4_ is a nanoscale construct with the silver molecule buried within its DNA host. Oligonucleotides are synthetic platforms as specific nucleobases and even specific heteroatoms in nucleobases can be targeted to elicit large swings in the spectra and brightness of a DNA-bound cluster. We are investigating whether these same chemical and biochemical tools can be harnessed to guide DNA–silver molecule photochemistry.

## Conclusions

C_4_AC_4_TC_3_GT_4_ develops a binding pocket that protects and controls the photoreactivity of Ag_10_^6+^. Our studies highlighted two features of this DNA-caged reaction. DNA hinders photooxidation by encapsulating and protecting the cluster from exogenous O_2_. Also, the single Ag_10_^6+^ adduct degrades to the series Ag_6_^4+^, Ag_7_^5+^, Ag_8_^6+^, and Ag_9_^7+^ that loosely fit into the DNA coordination site. By leveraging chemical and biochemical tools, a DNA cage might be reshaped to regulate the photochemical reactivity of DNA-encapsulated silver clusters.

## Supporting Information Available

Photodestruction quantum yields and isotopologue distributions for the silver adducts with C_4_AC_4_TC_3_GT_4_; excitation spectra of Ag_10_^6+^/C_4_AC_4_TC_3_GT_4_ using λ_em_ = 534 and 700 nm; time evolution of the green emission and red luminescence using different emission filters; absorption spectra collected in H_2_O and D_2_O; time evolution of *A*_490_ based on data in Figure 2; isotopologue distribution for the −4 charged anion based on the molecular formula C_169_H_212_N_53_O_110_P_17_Ag_10_; mass spectra of Ag^+^–DNA complexes with initial stoichiometries of 2 Ag^+^:DNA, 4 Ag^+^:DNA, and 8 Ag^+^: DNA; absorption and fluorescence spectra before and after adding 2 eq I^–^:DNA; mass spectra of C_4_AC_4_TC_3_GT_4_/Ag_10_^6+^ following photolysis and then dialysis with 100× (top) and 10,000× (bottom) volumes of buffer; absorption spectra with I^–^, Br^–^, and Cl^–^; absorption spectra collected with O_2_ and N_2_ purging; Stern–Volmer plot derived from the near-infrared luminescence lifetimes with varying oxygen concentrations; and absorption spectra of C_4_AC_4_TC_3_GT_4_/Ag_10_^6+^ before (red) and after (blue) irradiation with methylene blue (PDF)
