# Supplementary material for: Breaching the Fortress: Photochemistry of DNA-Caged Ag106+
Source: J Phys Chem B. 2023 Dec 6;127(50):10851–60. doi: 10.1021/acs.jpcb.3c06358 (PMC10749453; doi:10.1021/acs.jpcb.3c06358)
Supplement: Supplementary file 1 — jp3c06358_si_001.pdf [file jp3c06358_si_001.pdf]

Breaching the Fortress: Photochemistry of DNA-Caged Ag_{10}^{6+}

Caleb J. Setzler,[§] Caleb A. Arrington,[‡], David Lewis[§], and Jeffrey T. Petty [§*]

[‡]Department of Chemistry, Wofford College, Spartanburg, SC 29303, United States
[§]Department of Chemistry, Furman University, Greenville, SC 29163, United States

Table S1: Example Calculations for Photodestruction Quantum Yield (Φ_d)[a]

Sample	*t (s)*	*P (W)*	A_l	N_{phot}	A_{490}	ΔA_{490}	N_{molec}	Φ_d
59EE05	0	0	1.187		1.2660			
59EE06	120	2.28E-03	1.169	6.37E+17	1.2490	0.0170	8.62E+13	1.35E-04
59EE07	120	2.24E-03	1.148	6.24E+17	1.2260	0.0230	1.17E+14	1.87E-04
59EE08	120	5.00E-03	1.11	1.38E+18	1.1780	0.0480	2.43E+14	1.76E-04
59EE09	120	5.00E-03	1.074	1.37E+18	1.1390	0.0390	1.98E+14	1.44E-04
59EE10	120	5.00E-03	1.042	1.36E+18	1.1010	0.0380	1.93E+14	1.41E-04
59EE11	120	5.00E-03	1.008	1.35E+18	1.0660	0.0350	1.77E+14	1.31E-04
59EE12	60	1.00E-02	0.978	1.34E+18	1.0310	0.0350	1.77E+14	1.32E-04
59EE13	600	1.00E-03	0.945	1.33E+18	0.9920	0.0390	1.98E+14	1.49E-04
59EE14	120	5.10E-03	0.914	1.34E+18	0.9600	0.0320	1.62E+14	1.21E-04
59EE15	120	5.10E-03	0.886	1.33E+18	0.9270	0.0330	1.67E+14	1.26E-04

λ_l (m)	4.965E-07
h (J/s)	6.626E-34
c (m/s)	3.00E+08

V_{sol} (L)	4.04E-04
b (cm)	1.00
ε (M^{-1} cm^{-1})	48,000
N_A (molec/mol)	6.02E+23

$$N_{phot} = \frac{P \cdot t \cdot \lambda \left(1 - \frac{1}{10^{A_l}}\right)}{h \cdot c}$$

$$N_{molec} = \frac{\Delta A_{490} \cdot V_{sol} \cdot N_A}{b \cdot \varepsilon}$$

[a] *t* is the irradiation time, *P* is the power, A_l is the absorbance at the laser wavelength (496.5 nm), N_{phot} is the number of photons absorbed, λ_l is the laser wavelength, *h* is Planks constant, *c* is the speed of light, A_{490} is the cluster absorbance at 490 nm, ΔA_{490} is the change in the absorbance at 490 nm, N_{molec} is the number of photobleached molecules, V_{sol} is the volume of the solution, *b* is the pathlength of the cell, ε is the extinction coefficient, and N_A is Avogadro's number.

Table S2: Isotopologue M/Z and Intensity Analysis for $(C_4AC_4TC_3GT_4/Ag_{10}^{6+})^{-4}$

Measure M/Z[a]	Measured Intensity[b]	Predicted M/Z[c]	Predicted Intensity[d]	$\Delta_{M/Z}$ (ppm)[e]	Δ_{Int}[f]
1585.9667	16.0%	1585.9677	12.7%	0.6	3.3%
1586.2163	30.4%	1586.2174	25.6%	0.7	4.9%
1586.4664	40.6%	1586.4678	36.0%	0.9	4.6%
1586.7162	61.2%	1586.7177	55.8%	0.9	5.4%
1586.9668	71.7%	1586.9679	69.1%	0.7	2.7%
1587.2164	89.8%	1587.2178	87.1%	0.9	2.7%
1587.4664	93.3%	1587.468	94.6%	1.0	1.2%
1587.7161	100.0%	1587.718	100.0%	1.2	0%
1587.9667	93.4%	1587.9681	95.1%	0.9	1.7%
1588.2162	87.7%	1588.2183	86.0%	1.3	1.7%
1588.4661	74.8%	1588.4684	71.4%	1.4	3.4%
1588.7156	62.4%	1588.7185	55.6%	1.8	6.8%
1588.9655	48.5%	1588.9688	40.2%	2.1	8.3%
1589.2153	36.3%	1589.2189	27.1%	2.3	9.2%
1589.4659	25.7%	1589.4691	17.0%	2.0	8.8%
1589.7158	17.3%	1589.7194	9.9%	2.3	7.4%
			Average:	1.3	4.5%

[a]Measured peak positions.
[b]Measured intensities relative to the most intense peak.
[c]Predicted peak position based on the formula $C_{169}H_{212}N_{53}O_{110}P_{17}Ag_{10}$.
[d]Predicted intensities based on natural isotopologue distributions
[e]Absolute M/Z differences between observed and predicted values relative to the observed peak position. These differences are reported as parts per million.
[f]Intensity differences between observed and predicted values.

Table S3: Isotopologue M/Z and Intensity Analysis for $(C_4AC_4TC_3GT_4/Ag_9^{7+})^{-4}$ [a]

Mesured M/Z	Mesured Intensity	Predicted M/Z	Predicted Intensity	ΔM/Z	ΔInt
1558.7406	18.8%	1558.7390	12.5%	1.0	6.3%
1558.9901	25.8%	1558.9895	19.6%	0.4	6.2%
1559.2410	41.3%	1559.2393	36.5%	1.1	4.8%
1559.4891	53.5%	1559.4895	49.6%	0.3	3.9%
1559.7384	74.3%	1559.7395	71.1%	0.7	3.1%
1559.9899	86.1%	1559.9896	83.9%	0.2	2.1%
1560.2394	99.3%	1560.2397	97.7%	0.2	1.6%
1560.4891	99.9%	1560.4899	100.0%	0.5	0.1%
1560.7388	100.0%	1560.7400	97.3%	0.8	2.7%
1560.9894	90.1%	1560.9901	86.1%	0.4	4.0%
1561.2396	78.3%	1561.2402	71.3%	0.4	7.0%
1561.4900	62.1%	1561.4904	54.3%	0.3	7.8%
1561.7405	47.6%	1561.7406	38.4%	0.1	9.2%
1561.9911	33.8%	1561.9908	25.2%	0.2	8.7%
1562.2440	23.2%	1562.2410	15.2%	1.9	8.0%
			Average:	0.6	5.0%

[a]See footnotes for Table S2. Peak positions based on the formula $C_{169}H_{211}N_{53}O_{110}P_{17}Ag_9$.

Table S4: Isotopologue M/Z and Intensity Analysis for $(C_4AC_4TC_3GT_4/Ag_8^{6+})^{-4}$ [a]

Mesured M/Z	Mesured Intensity	Predicted M/Z	Predicted Intensity	ΔM/Z	ΔInt
1532.2643	22.3%	1532.2648	18.8%	0.3	3.5%
1532.5148	32.3%	1532.5151	28.6%	0.2	3.7%
1532.7645	53.4%	1532.7650	49.0%	0.3	4.4%
1533.0143	65.7%	1533.0153	63.8%	0.7	1.9%
1533.2639	86.2%	1533.2653	84.1%	0.9	2.2%
1533.5143	92.6%	1533.5155	93.7%	0.8	1.1%
1533.7640	100.0%	1533.7655	100.0%	1.0	0.0%
1534.0143	94.8%	1534.0157	95.2%	0.9	0.3%
1534.2643	87.5%	1534.2659	84.6%	1.0	2.9%
1534.5139	73.3%	1534.5160	68.6%	1.4	4.8%
1534.7632	59.3%	1534.7662	51.3%	2.0	8.0%
1535.0133	44.3%	1535.0164	35.3%	2.0	9.0%
1535.2635	31.2%	1535.2666	22.4%	2.0	8.8%
1535.5142	20.5%	1535.5168	13.0%	1.7	7.5%
			Average:	1.1	4.1%

[a]See footnotes for Table S2. Peak positions based on the formula $C_{169}H_{212}N_{53}O_{110}P_{17}Ag_8$.

Table S5: Isotopologue M/Z and Intensity Analysis for $(C_4AC_4TC_3GT_4/Ag_7^{5+})^{-4}$ [a]

Mesured M/Z	Mesured Intensity	Predicted M/Z	Predicted Intensity	ΔM/Z	ΔInt
1505.5382	16.5%	1505.5408	12.6%	1.7	3.9%
1505.7883	32.8%	1505.7905	28.4%	1.5	4.4%
1506.0400	45.9%	1506.0409	41.6%	0.6	4.3%
1506.2894	68.8%	1506.2908	65.0%	0.9	3.8%
1506.5396	81.9%	1506.5410	80.2%	0.9	1.7%
1506.7891	97.6%	1506.7911	96.4%	1.3	1.2%
1507.0404	100.0%	1507.0413	100.0%	0.6	0.0%
1507.2897	98.3%	1507.2914	96.9%	1.1	1.3%
1507.5391	88.0%	1507.5416	84.5%	1.7	3.5%
1507.7888	74.4%	1507.7917	67.6%	1.9	6.8%
1508.0392	58.0%	1508.0420	49.3%	1.9	8.7%
1508.2892	41.8%	1508.2922	32.9%	2.0	8.9%
1508.5389	28.4%	1508.5425	20.0%	2.4	8.4%
1508.7896	18.3%	1508.7928	11.2%	2.1	7.1%
			Average:	1.5	4.6%

[a]See footnotes for Table S2. Peak positions based on the formula $C_{169}H_{213}N_{53}O_{110}P_{17}Ag_7$.

Table S6: Isotopologue M/Z and Intensity Analysis for $(C_4AC_4TC_3GT_4/Ag_6^{4+})^{-4}$ [a]

Mesured M/Z	Mesured Intensity	Predicted M/Z	Predicted Intensity	ΔM/Z	ΔInt
1478.8134	14.5%	1478.816	11.7%	1.8	2.8%
1479.0648	23.2%	1479.0665	20.0%	1.1	3.2%
1479.3152	44.8%	1479.3163	40.4%	0.7	4.3%
1479.5649	59.7%	1479.5667	56.8%	1.2	3.0%
1479.8149	82.8%	1479.8167	80.0%	1.2	2.8%
1480.0654	92.4%	1480.0669	92.4%	1.0	0.0%
1480.3151	100.0%	1480.3169	100.0%	1.2	0.0%
1480.5643	96.1%	1480.5671	95.1%	1.9	1.0%
1480.8141	86.8%	1480.8173	82.5%	2.2	4.3%
1481.0653	71.7%	1481.0675	64.5%	1.5	7.2%
1481.3134	54.9%	1481.3177	45.7%	2.9	9.2%
1481.5625	39.6%	1481.5681	29.3%	3.8	10.3%
1481.8119	26.5%	1481.8184	17.1%	4.4	9.4%
1482.0646	16.4%	1482.0687	9.0%	2.8	7.3%
			Average:	2.0	4.6%

[a]See footnotes for Table S2. Peak positions based on the formula $C_{169}H_{214}N_{53}O_{110}P_{17}Ag_6$.

Table S7: Summary of Isotopologue Distributions for $C_4AC_4TC_3GT_4/Ag_{10}^{6+}$ with -4, -5, -6, and -7 Net Charges

Overall Charge[a]	Cluster Charge[b]	Δ_{Int}[b]	$\Delta_{M/Z}$[c]
-4	**+6**	**4.5%**	**1.3**
	+7	12.1%	
	+5	9.7%	
-5	**+6**	**3.6%**	**4.9**
	+7	14.1%	
	+5	7.9%	
-6	**+6**	**1.8%**	**1.6**
	+7	12.3%	
	+5	10.0%	
-7	**+6**	**5.8%**	**9.6**
	+7	17.2%	
	+5	6.3%	

[a]Net charge of the $C_4AC_4TC_3GT_4/Ag_{10}^{6+}$ complexes
[b]Three models with Ag_{10}^{6+}, Ag_{10}^{6+}, and Ag_{10}^{6+} were considered. The Ag_{10}^{6+} gave the smallest standard deviation for the intensities and is bolded for emphasis.
[c]Absolute M/Z differences between observed and predicted values. These differences are reported in ppm.

Table S8: Summary of Silver Cluster Adducts with $C_4AC_4TC_3GT_4$

Adduct	$\overline{\Delta}_{Int}$[c]	$\overline{\Delta}_{M/Z}$[b]
Ag_{10}^{6+}	5.4%	4.4
Ag_9^{7+}	5.6%	2.4
Ag_8^{6+}	5.8%	4.5
Ag_7^{5+}	6.0%	5.1
Ag_6^{4+}	7.7%	7.8
6 Ag^+	7.7%	5.9
5 Ag^+	7.3%	5.2
4 Ag^+	7.4%	4.8
3 Ag^+	8.7%	6.5
2 Ag^+	8.8%	5.4
1 Ag^+	21.1%	8.2
0 Ag^+	14.2%	5.5

[a]Average intensity differences between observed and predicted values for 3-4 charge states.

[b]Average M/Z differences between observed and predicted values for 3-4 charge states.

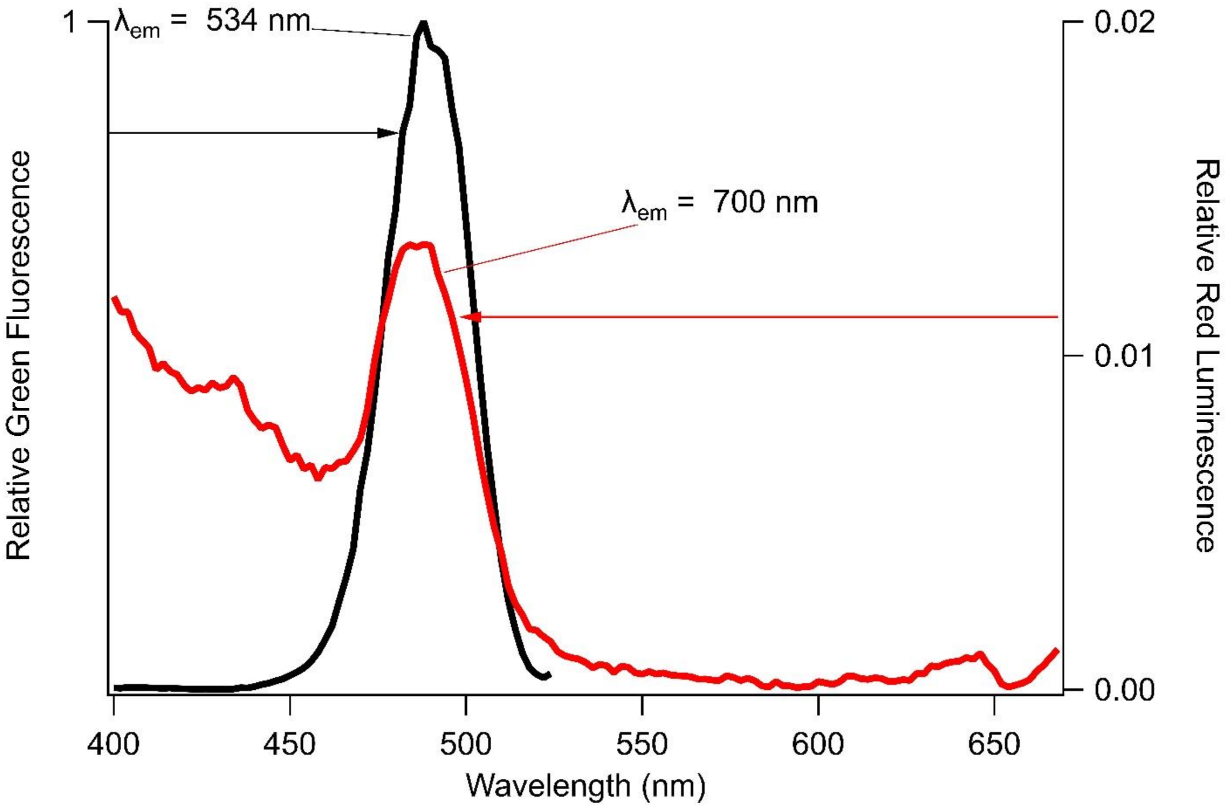

Figure S1: Excitation spectra of $Ag_{10}^{6+}/C_4AC_4TC_3GT_4$ using λ_{em} = 534 (black) and 700 (red) nm emission acquired at 77 K in a cryogenic ethylene glycol/buffer matrix. Coincident peaks suggest that the chromophore is excited to its green-emissive S_1 state and then relaxes to its lower-energy red luminescent L state (see Figure 1B). The red luminescence is ~100 fold weaker than the green fluorescence. The signal at higher energies is attributed to scattering from the cryogenic matrix.

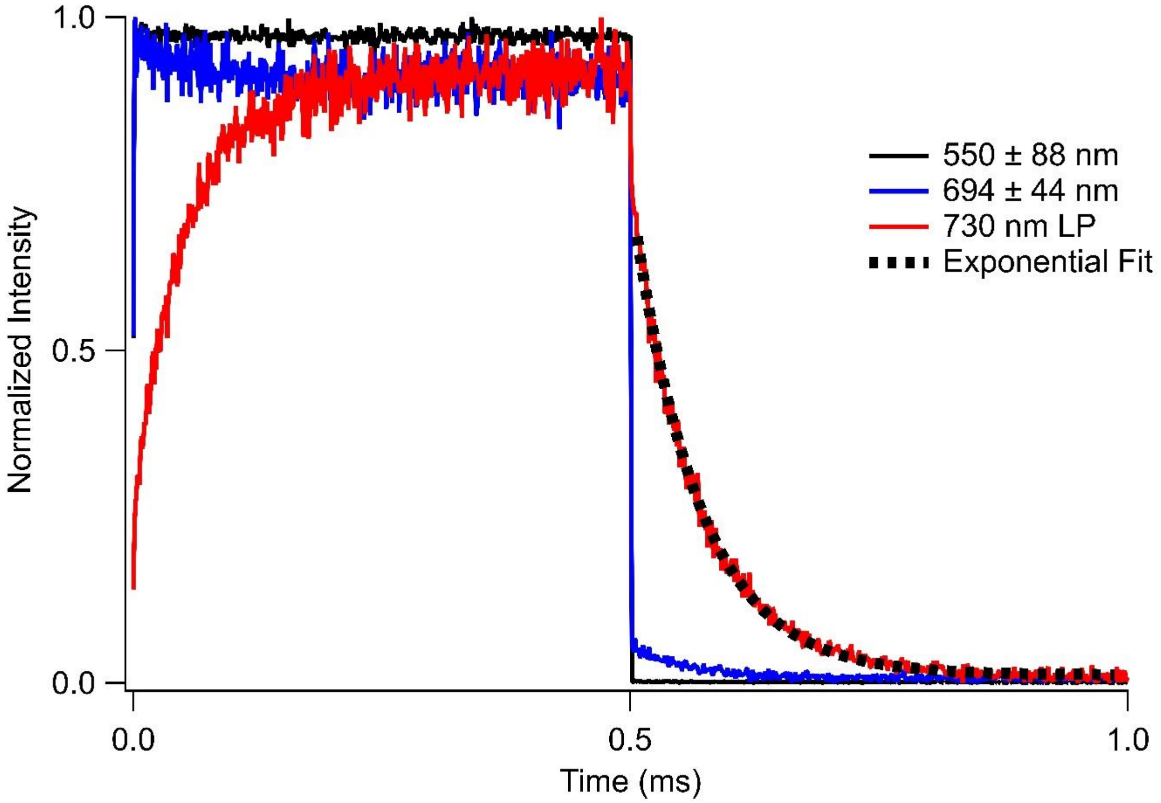

Figure S2: Time evolution of the green emission and red luminescence using different emission filters. The laser was modulated at 1 kHz with a 50% duty cycle. A 550 ± 88 nm filter (black) shows that the green emission promptly rises and falls, consistent with rapid $S_1 \rightarrow S_0$ fluorescence. As the spectral window is red-shifted to 694 ± 44 (blue) and 730 nm longpass (red), the red luminescence is distinguished because it more slowly rises and falls, consistent with disfavored transitions $S_1 \rightarrow L$ and $L \rightarrow S_0$, respectively (see Figure 1B). An exponential fit of the 730 nm LP data gives a lifetime of 66 μs.

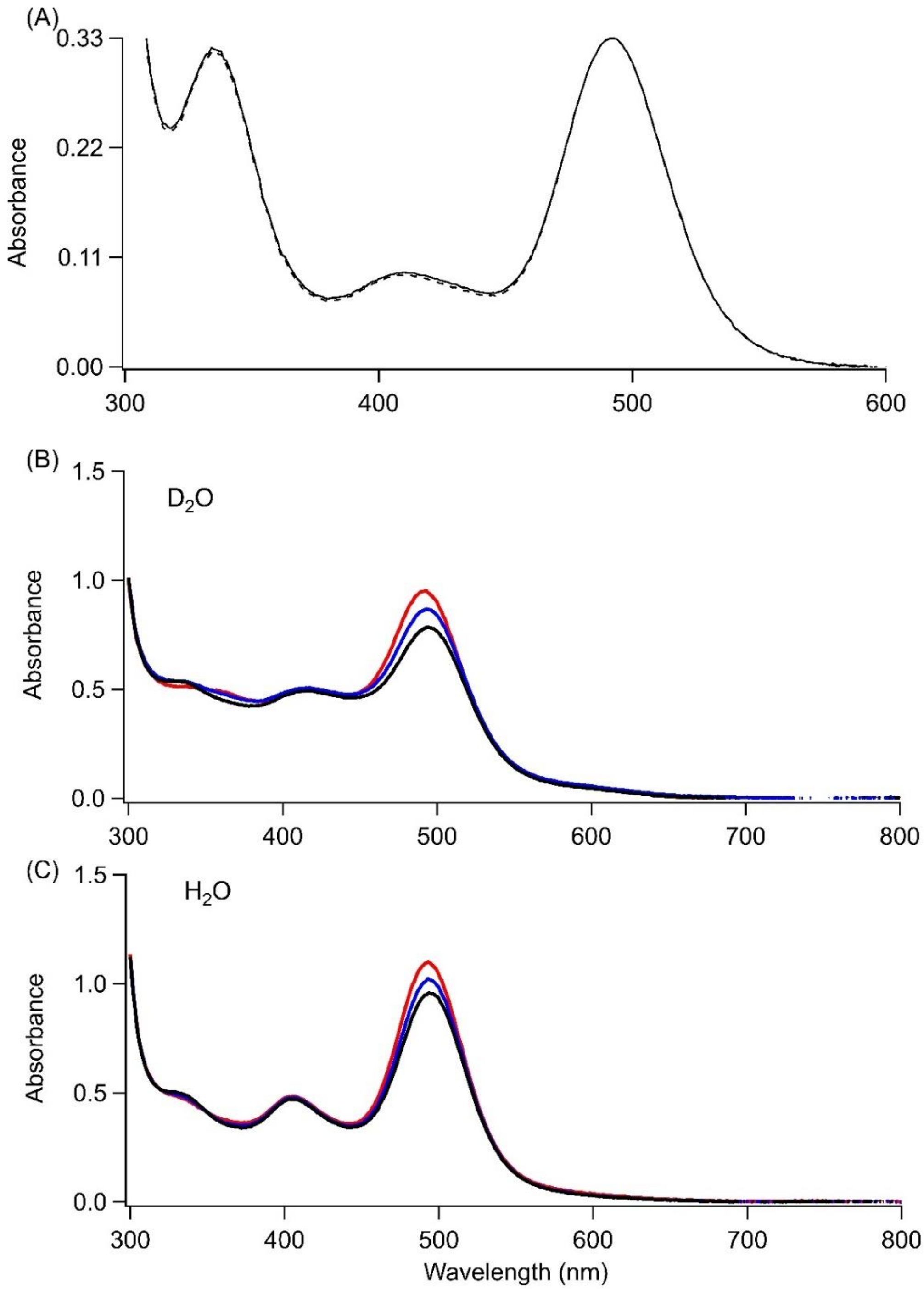

Figure S3: (A) An intermediate pair of absorption spectra extracted from Figure 2 before (solid) and after (dashed) waiting 5 minutes without irradiation. Overlapping spectra suggests that photoreaction stops without irradiation. (B/C) Absorption spectra during photoirradiation of Ag_{10}^{6+} clusters at λ = 496.5 nm in (B) D_2O and (C) H_2O. The red trace is before irradiation, the blue trace is after 15 minutes of photoirradiation with 2.3 mW, and the black trace is after an additional 10 minutes with 3.5 mW. The clusters degrade similarly, suggesting that the encapsulating DNA host protects its Ag_{10}^{6+} cluster.

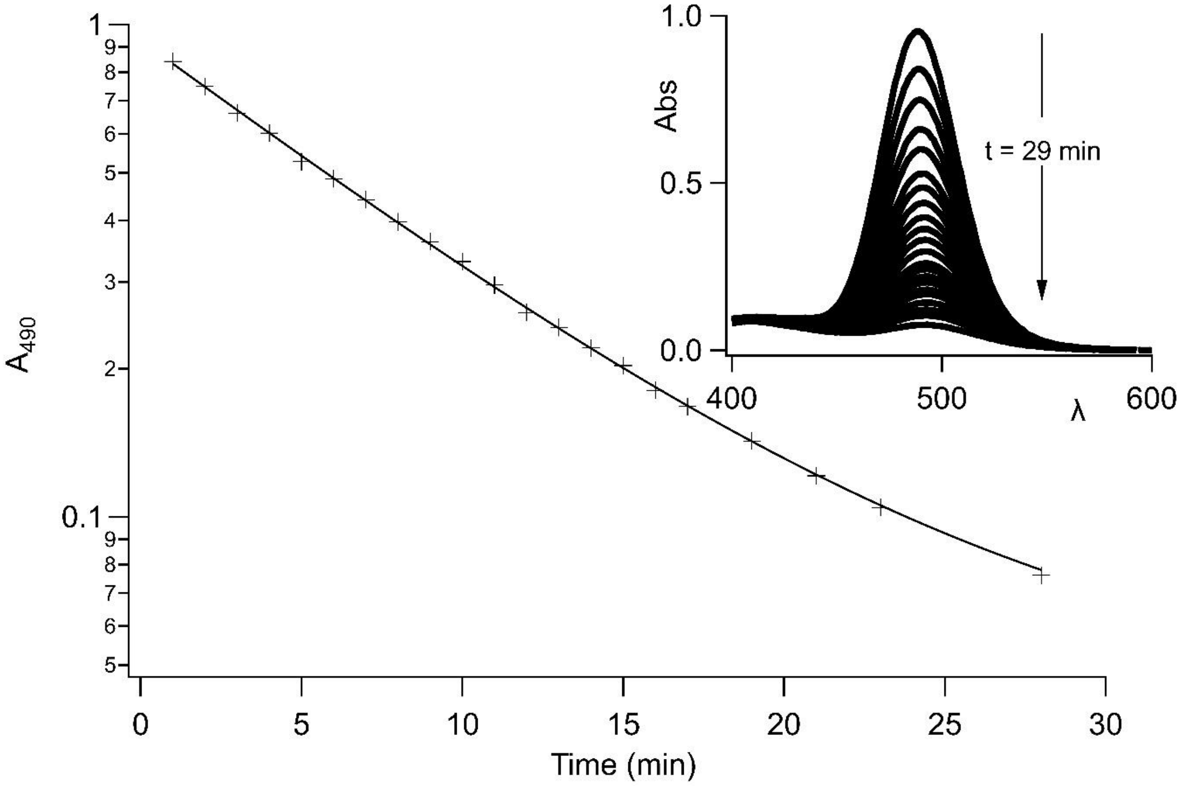

Figure S4: Time evolution of A_{490} based on data in Figure 2 (see inset). The data was fit using an exponential model, and the linear trend using a logarithmic scale supports a first-order or pseudo first-order reaction.

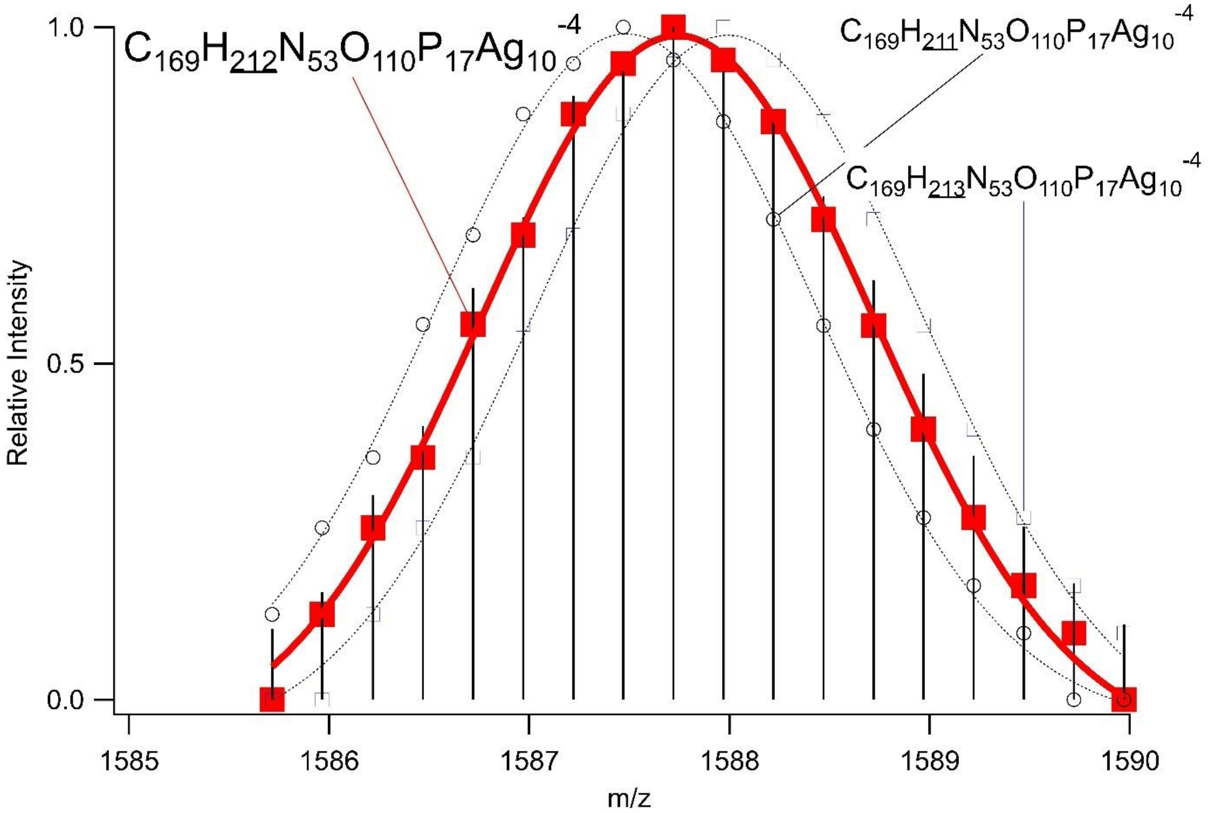

Figure S5: Isotopologue distributions for -4 charged DNA-Ag_{10}^{6+} anions based on the molecular formula $C_{169}H_{\underline{212}}N_{53}O_{110}P_{17}Ag_{10}$. Alternative models with ± 1 H^+ with respective formulas $C_{169}H_{\underline{211}}N_{53}O_{110}P_{17}Ag_{10}$ (open circles) and $C_{169}H_{\underline{213}}N_{53}O_{110}P_{17Ag10}^{6+}$ (open squares) have distributions that are statistically different based on the standard deviations for the intensities. These standard deviations are 4.5%, 12.1%, and 9,7% for the respective formulas with H_{212}, H_{211}, and H_{213}. Based on the smaller deviation for H_{212}, the cluster is assigned to be Ag_{10}^{6+} with a +6 oxidation state.

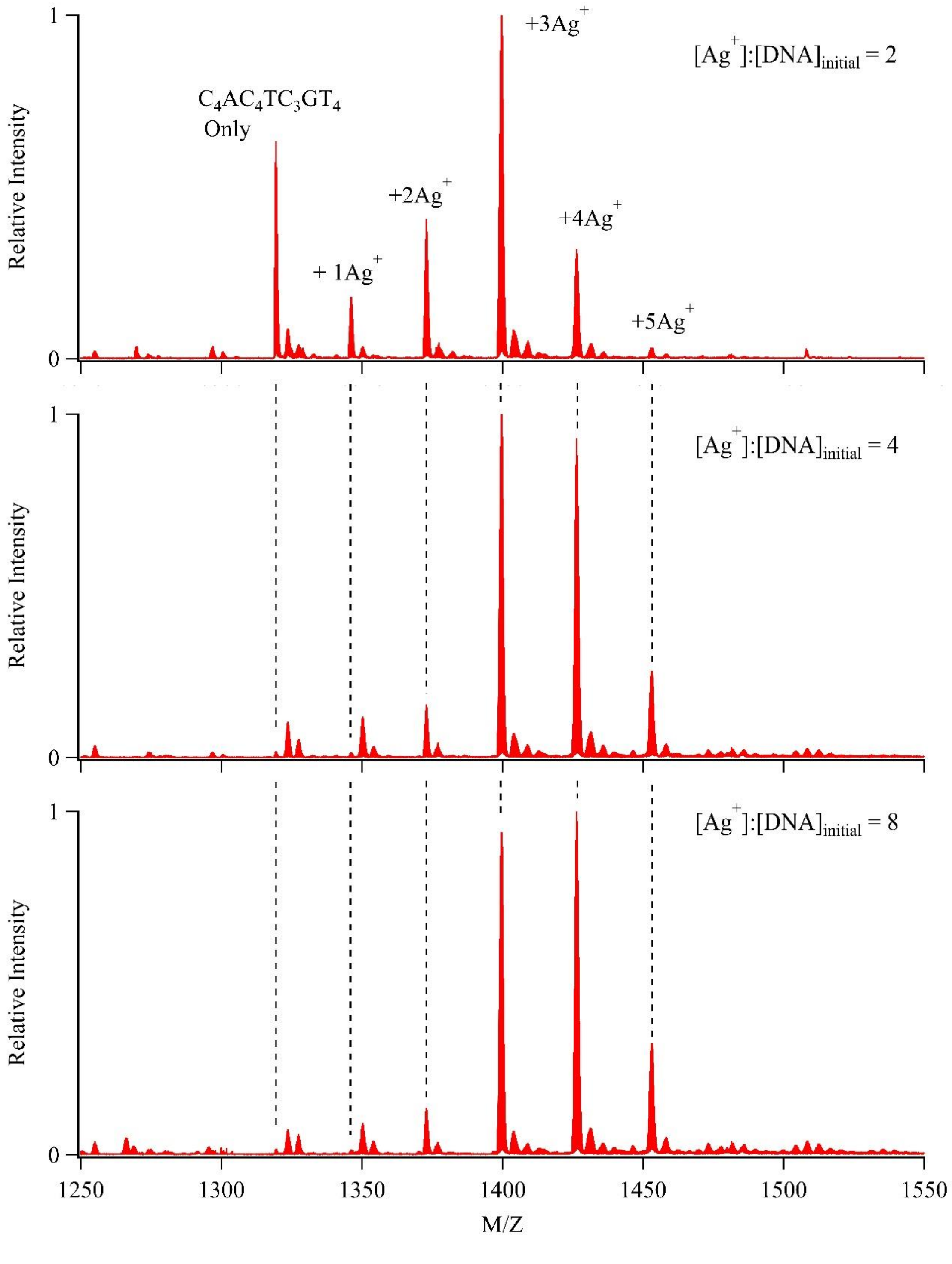

Figure S6: Mass spectra of Ag^+-DNA complexes with initial stoichiometries of 2 Ag^+:DNA, 4 Ag^+:DNA, and 8 Ag^+: DNA (top to bottom). These were diluted with 100x volumes of 5 mM ammonium acetate to dissociate weaker adducts. Similar distributions for the three spectra suggest that 3-4 Ag^+:DNA strongly bind with this DNA.

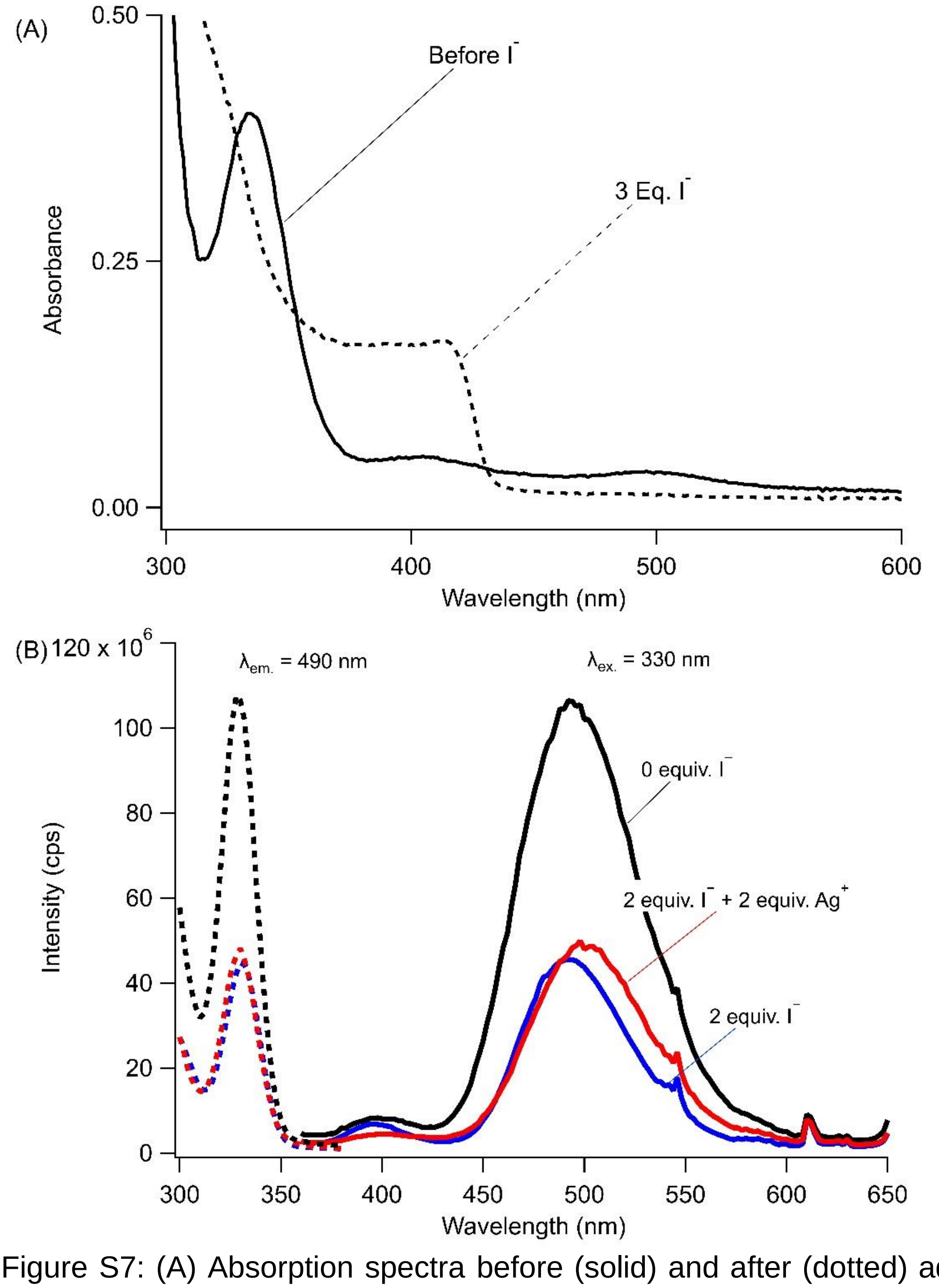

Figure S7: (A) Absorption spectra before (solid) and after (dotted) adding 3 Eq I^-:DNA. Absorption develops at λ ~ 420 nm, supporting precipitation of AgI(s). (B) Fluorescence excitation (dashed) and emission (solid) spectra acquired in an ethylene glycol/buffer matrix at 77K. The spectra were collected before (black), after adding 2 Eq. I^-:DNA (blue), and after replenishing the precipitated Ag^+ by adding 2 Eq. Ag^+:DNA (red). The fluorescence drops after adding I^-, which suggests that the larger clusters degrade. The fluorescence does not recover after adding Ag^+, which suggests that the Ag_6^{4+} is converted to larger clusters. The emission red shifts ~6 nm after adding 2 equivalents of Ag^+, which may be due to a change in the cluster environment.[1]

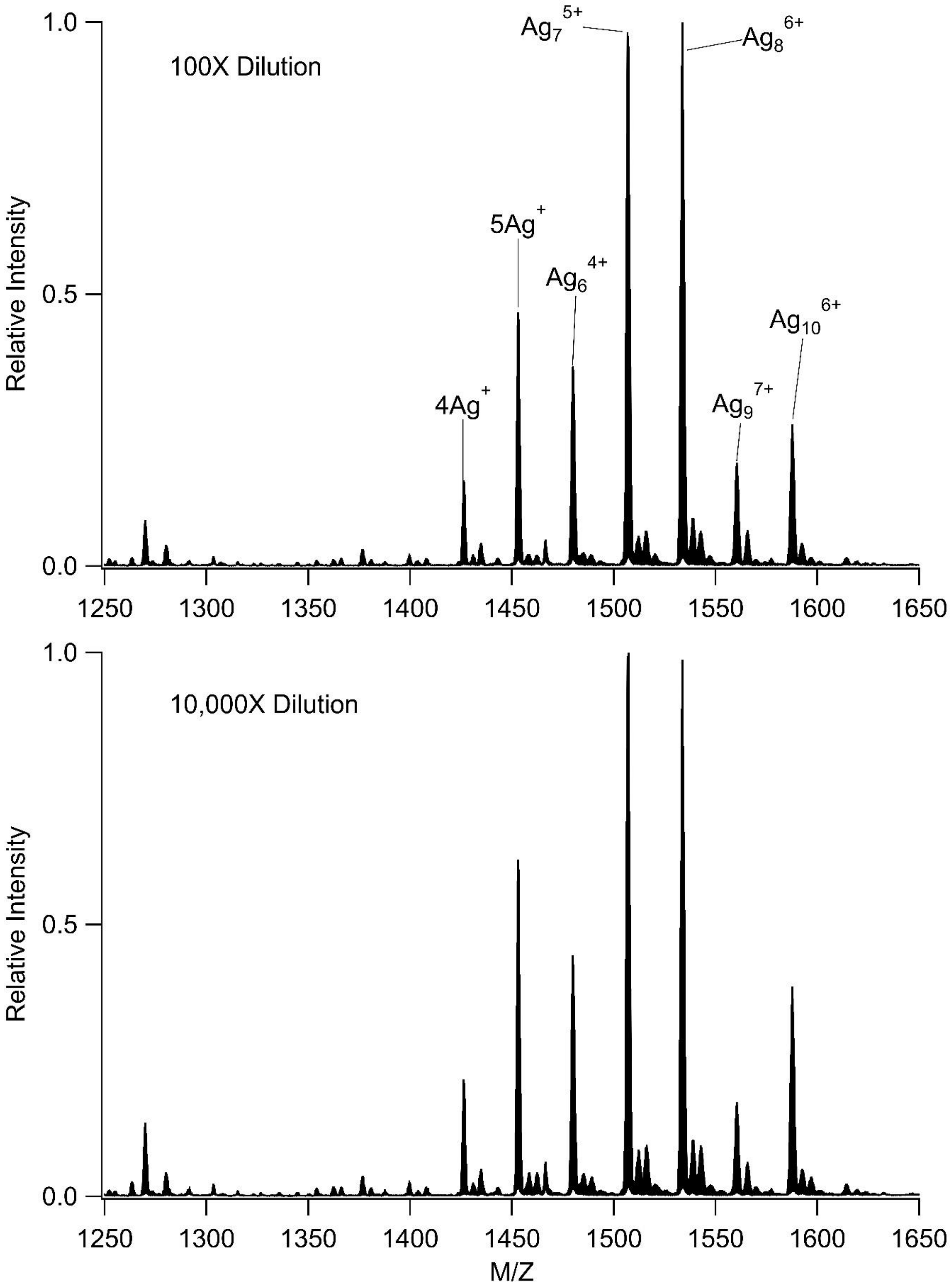

Figure S8: Mass spectra of $C_4AC_4TC_3GT_4/Ag_{10}^{6+}$ following photolysis and then dialysis with 100x (top) and 10000x (bottom) volumes of buffer. No significant change in the distribution of adducts suggests that all silvers are strongly bound.

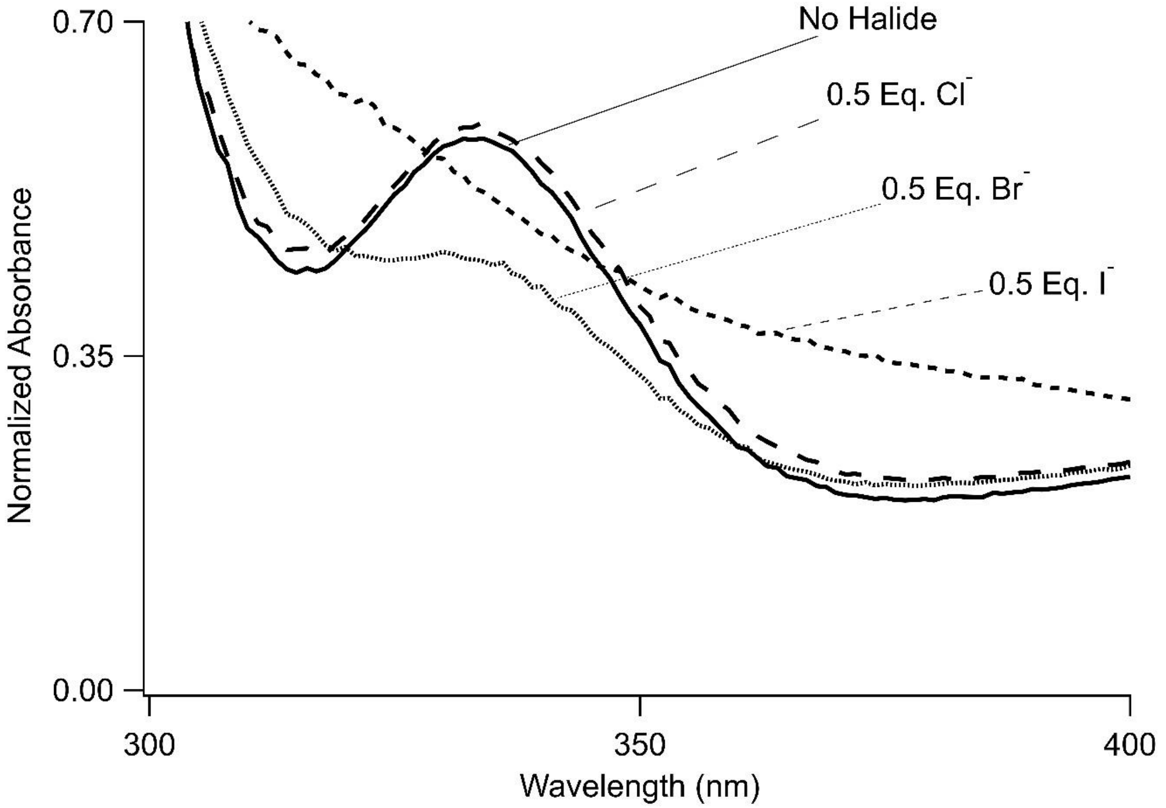

Figure S9: Absorption spectra with no halide (solid), with I^- (dashed), with Br^- (dotted), and Cl^- (long dashes). The amounts are 0.5 equivalents halide:DNA. Similar results without and with Cl^- suggest the Ag^+/Cl^- affinity is too low to precipitate AgCl(s).

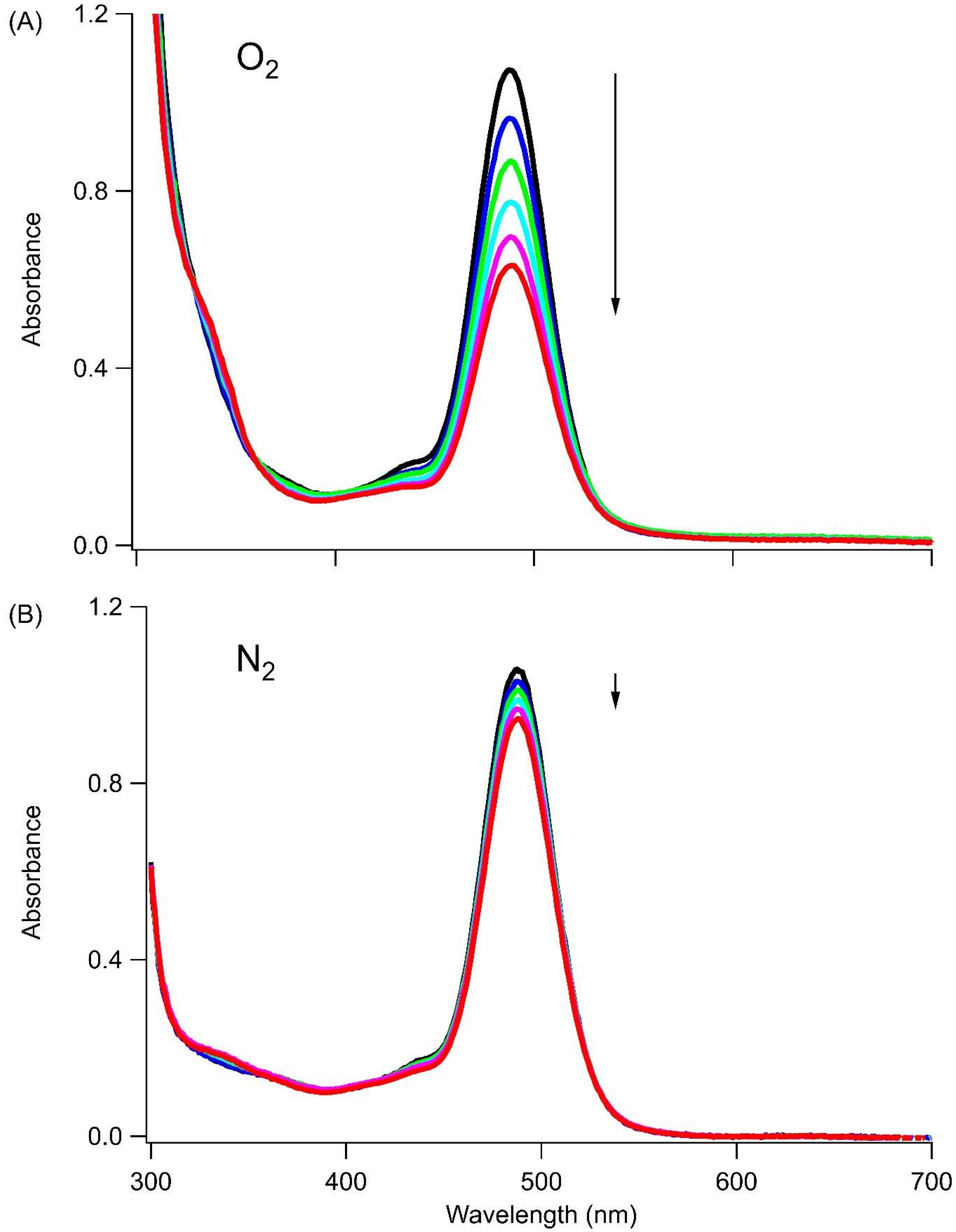

Figure S10: Absorption spectra collected before (black) and then in 1 min intervals using 1 mW at 490 nm (blue, green, aqua, magenta, and red, respectively). The arrows emphasize a more efficient reaction with O_2 vs N_2.

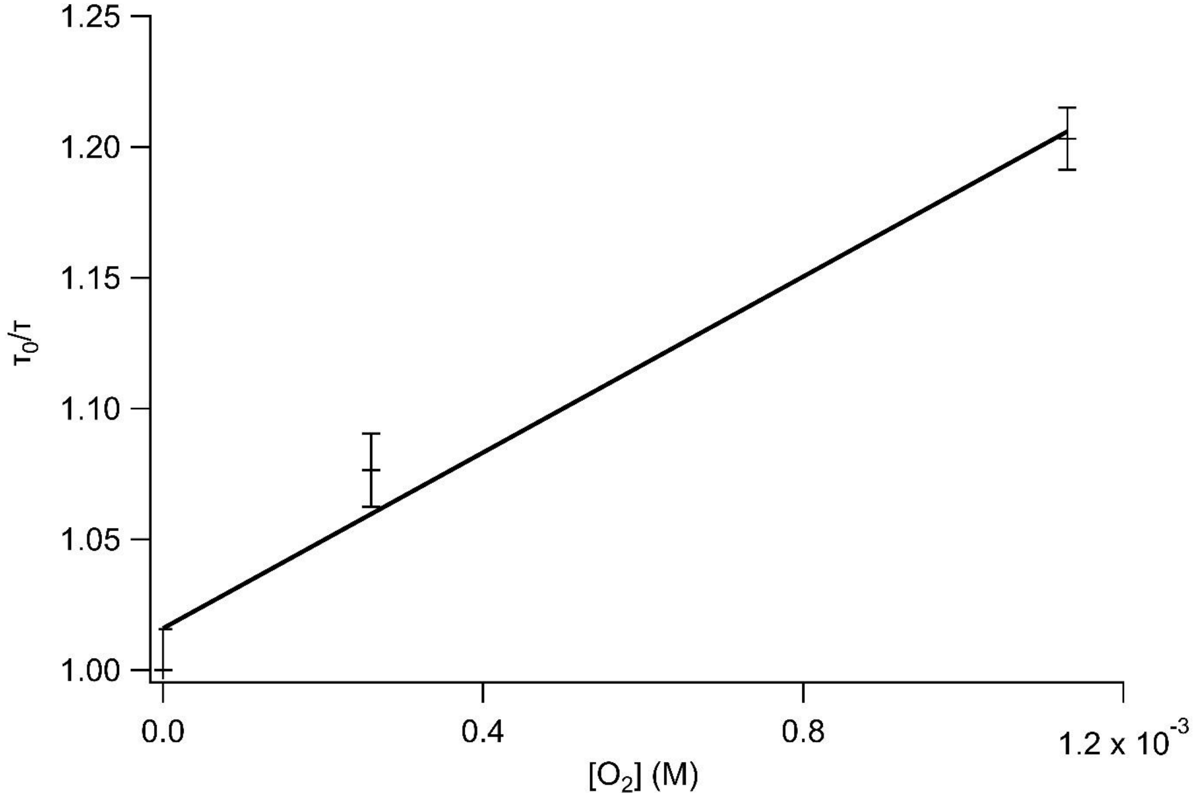

Figure S11: Stern–Volmer plot derived from the luminescence lifetimes without (τ_0) and with (τ) oxygen ($[O_2]$). The slope $\tau_0 k_{SV}$ from the linear fit yields k_{SV} = 2.3 (±0.2) × 10^6 M^{-1} s^{-1}.

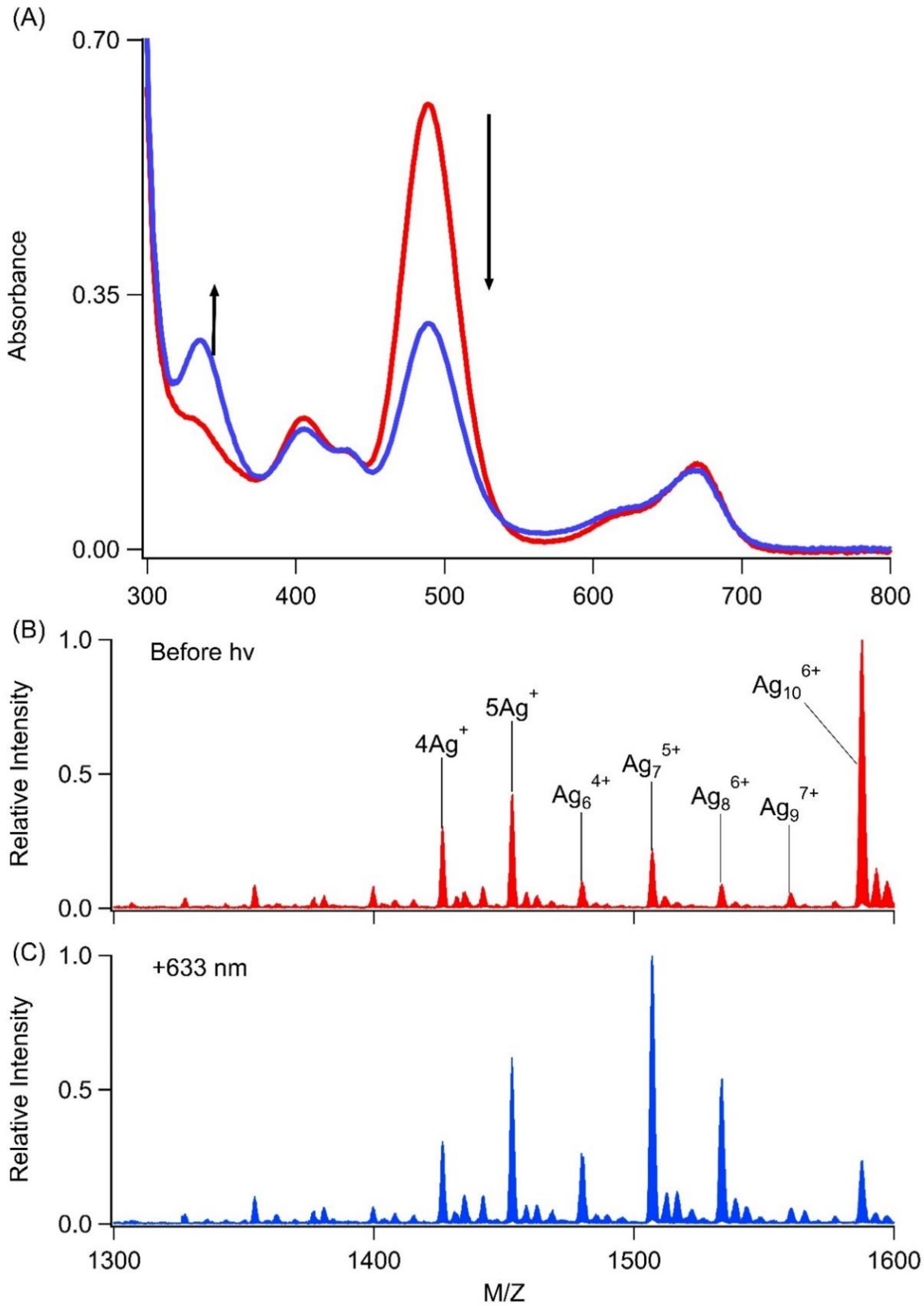

Figure S12: (A) Absorption spectra of $C_4AC_4TC_3GT_4/Ag_{10}^{6+}$ before (red) and after (blue) irradiation with methylene blue at 632.8 nm. The λ ~ 490 nm absorption falls while the l ~335 nm absorption grows with irradiation, while the λ ~ 630 nm absorption of methylene blue is relatively consistent. (B and C) As observed by direct Ag_{10}^{6+} irradiation, mass spectra before and after irradiation shows the same loss of $C_4AC_4TC_3GT_4/Ag_{10}^{6+}$ and growth of the more oxidized products.

(1) Yeh, H.-C.; Sharma, J.; Shih, I.-M.; Vu, D. M.; Martinez, J. S.; Werner, J. H., A Fluorescence Light-up Ag Nanocluster Probe That Discriminates Single-Nucleotide Variants by Emission Color. *J. Am. Chem. Soc.* **2012**, *134*, 11550-11558.
